# Electrocatalytic Self-Coupling of N-Heterocyclic Amides for Energy-Efficient Bipolar Hydrogen Production

**DOI:** 10.1007/s40820-025-02025-3

**Published:** 2026-01-04

**Authors:** Yuqiang Ma, Meng Li, Dandan Zhang, Cihang Wang, Yu Li, Zihang Zhao, Xiaogang Mu, Jun Hu, Xiang Hu, Jiachen Li, Haixia Ma, Zhenhai Wen

**Affiliations:** 1 Xi’an Key Laboratory of Special Energetic Materials, School of Chemical Engineering, Northwest University, Xi’an, 710127 People’s Republic of China; 2 Zhijian Laboratory, Xi’an, 710025 People’s Republic of China; 3 State Key Laboratory of Structural Chemistry, and Fujian Provincial Key Laboratory of Materials and Techniques Toward Hydrogen Energy, Fujian Institute of Research On the Structure of Matter, Chinese Academy of Sciences, Fuzhou, Fujian 350002 People’s Republic of China

**Keywords:** Bipolar hydrogen production, Electrosynthesis, Coupling system, 5,5′-diamino-3,3′-azo-1H-1,2,4-triazole, Pt-based catalyst

## Abstract

This study proposes a green electrochemical strategy for addressing the high-energy-barrier oxygen evolution reaction (OER) in traditional overall water splitting. Leveraging the thermodynamic advantages of N–H bond activation/cleavage and N–N coupling processes, the 3,5-diamino-1,2,4-triazole (DAT) oxidative coupling reaction (DATOR) has been introduced to replace the high-energy-barrier oxygen evolution reaction (OER). This substitution enables low-energy-consumption hydrogen production while simultaneously yielding high-value azo energetic materials. Furthermore, to enhance electron and atom economy, the anodic DATOR process allows the hydrogen radicals (H*) generated from amine dehydrogenation to chemically combine via the Tafel process, producing hydrogen gas. By constructing coupling system with Pt_s,n_@NiS_2_@CC cathode and CuO/CF anode, the operating voltage of the system was significantly reduced (0.96 V@10 mA cm^− 2^), which was 680 mV more energy efficient than conventional water electrolysis (1.64 V). In situ spectroscopy and theoretical calculations indicate that the anode DATOR generates DAAT through the N–H bond cleavage and N–N coupling path mediated by hydroxyl radicals (OH*), while releasing hydrogen gas. The coupling system has been operating stably for more than 300 h at an industrial-grade current density. This research provides new ideas for dual-electrode hydrogen production and green electrosynthesis of functional materials, with significant energy and economic benefits.

**Supplementary Information:**

The online version contains supplementary material available at 10.1007/s40820-025-02025-3.

## Highlights

- Replacing anodic oxygen evolution reaction with 3,5-diamino-1,2,4-triazole oxidative coupling enables ultra-low-voltage (0.96 V @10 mA cm^− 2^) dual-electrode H_2_ production and simultaneous synthesis of energetic 5,5′-diamino-3,3′-azido-1H-1,2,4-triazole (DAAT), achieving 35.8% energy savings.
- A Pt single-atom/nanoparticle hybrid on NiS_2_ nanosheets (Pt_s,n_@NiS_2_@CC) exhibits exceptional alkaline hydrogen evolution reaction performance and stability via optimized H* adsorption.
- Anodic DAAT formation proceeds via an OH*-mediated N–N coupling pathway, enabling stable (> 300 h @500 mA cm^− 2^), industrial-scale bipolar H_2_ production coupled with green DAAT synthesis in an anion-exchange membrane water electrolyzer.

**Supplementary Information:**

The online version contains supplementary material available at 10.1007/s40820-025-02025-3.

## Graphical abstract

**Figure:**
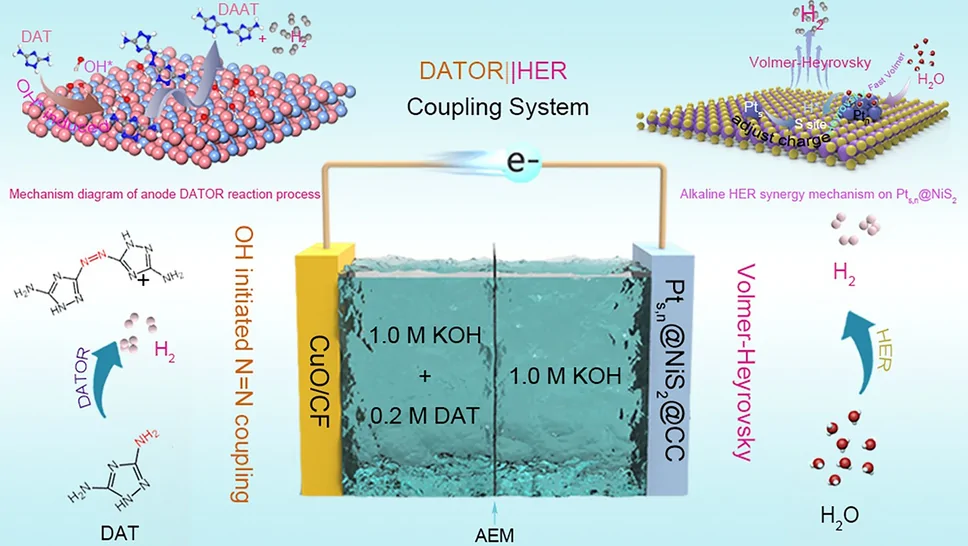

## Introduction

Hydrogen is considered a promising energy source to replace fossil fuels in the future [1–3]. Among them, hydrogen production by electrolysis of water is an environmentally friendly and efficient means of hydrogen production [4–6]. At present, hydrogen production from water electrolysis has not been applied on a large scale, the fundamental reason being that the slow oxygen precipitation reaction results in the need for water electrolysis to be carried out at high cell voltages (> 1.7 V) [7–9]. Therefore, reducing power consumption and designing efficient and inexpensive catalysts are key to reducing the cost of water decomposition [10–12]. Many researchers have worked on developing cost-effective catalysts for cost reduction and efficiency [13]. However, high-efficiency catalysts still cannot break through the thermodynamic barrier limitation of the oxygen evolution reaction (OER) [14–16]. To address this limitation, a proven method is to replace the anode OER with a reaction that thermodynamically has a low oxidation potential [17–19]. Notably, coupled systems of HER and small molecule oxidation have been extensively studied in recent years, with substrate molecules mainly including alcohols [20–22], 5-hydroxyfurfural [23, 24], formate [25], and urea [26, 27] used to enhance the value of the anode product [28]. Although significant results have been achieved with coupled systems, the market size mismatch between cathodic hydrogen production and anodic organic oxidation reactions still needs to be addressed [29]. The dual-electrode hydrogen production strategy is proposed to overcome the above problems. Wang et al. reported a dual-electrode hydrogen production system in which hydrogen is produced simultaneously at the cathode and anode at a low cell voltage of ~ 0.1 V. Instead of generating it directly based on water electrolysis, the system was realized by coupling low-potential anodic oxidation of biomass-derived aldehydes with cathodic hydrogen evolution reaction (HER) [30].

**Scheme 1:**
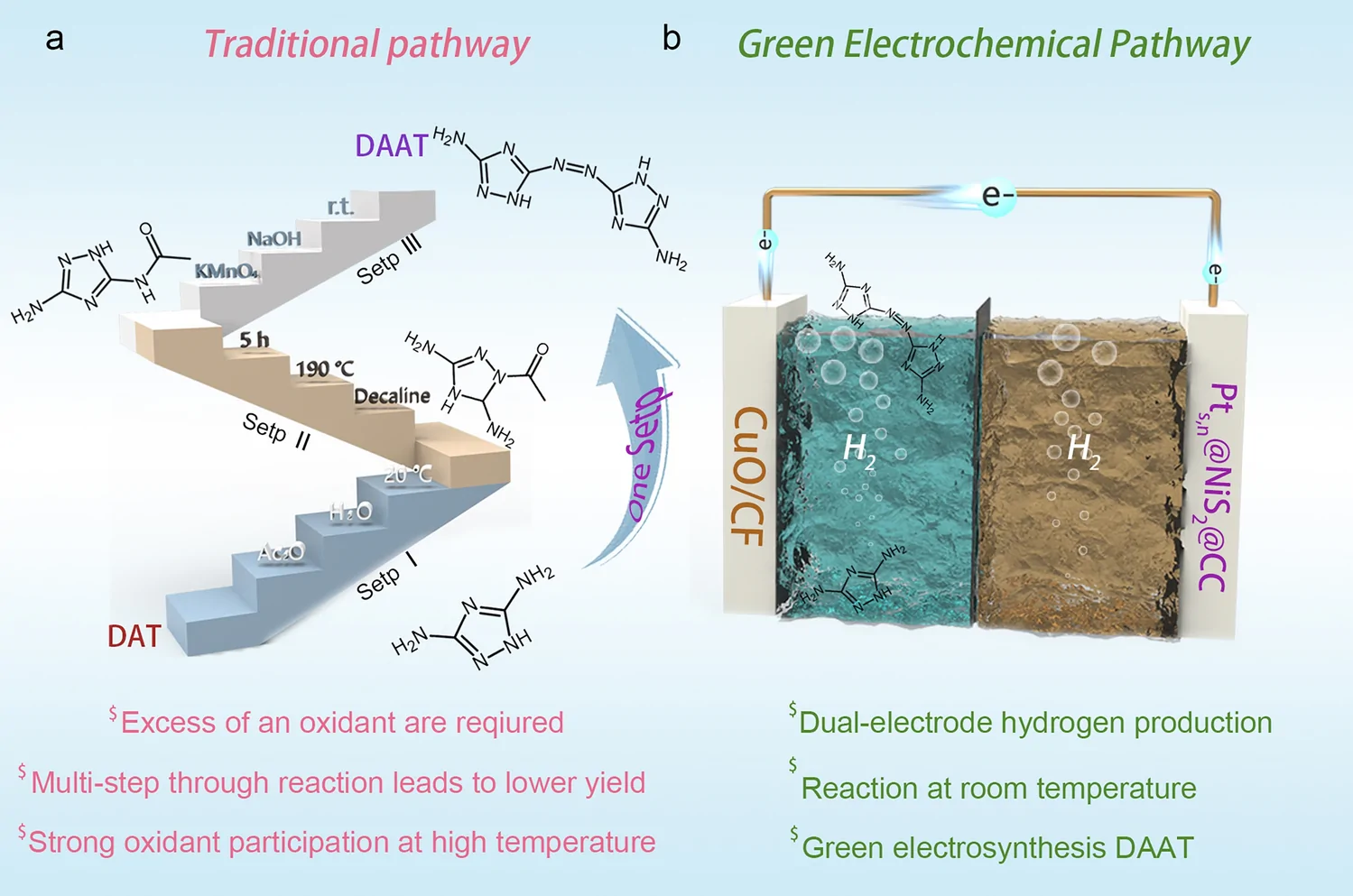
Schematic diagram of **a** the general preparation method of 5,5′-diamino-3,3′-azo-1H-1,2,4-triazole and **b** coupling system diagram

Azo compounds are used in a variety of fields due to their good thermal stability, positive oxygen balance, etc. Azo compounds with high-nitrogen content (e.g., azotriazole) are used as propellants or explosives [31]. However, the traditional chemical synthesis of azo compounds often requires the participation of strong oxidizing agents and high reaction temperatures, which increases the risk of the preparation process [32]. In addition, the reaction is often accompanied by the occurrence of side reactions, and subsequent means of purification increase the cost of preparation (Scheme 1a). Electrochemical azotization avoids the involvement of hazardous reagents and complex reaction processes. More importantly, azo compounds are usually obtained by self-coupling of amino compounds, a process that involves the breaking of the N–H bond accompanied by the release of H_2_ and has the potential to be used as a dual-electrode hydrogen production substrate [33]. Electrochemical conversion provides a gentler and more efficient method of obtaining organic products in the absence of additional reducing/oxidizing agents [34]. As for selective electrooxidation in aqueous solutions, the key challenge is the difficulty in controlling competing OER and other side reactions [35]. It is noteworthy that the N–N oxidative coupling in the DATOR process is thermodynamically more favorable (~ 0.5 V vs. RHE) than the OER (1.23 V vs. RHE) [36, 37]. Meanwhile, the H* generated after the N–H bond cleavage generates H_2_ by Tafel reaction under the action of electric current, realizing the anode hydrogen production [38]. Therefore, in this study, 5,5′-diamino-3,3′-azido-1H-1,2,4-triazole (DAAT) compounds were synthesized by one-step oxidative coupling dehydrogenation reaction via electrochemical reaction using 3,5-diamino-1,2,4-triazole (DAT) as a substrate, and H_2_ was generated simultaneously at the poles. The conventional synthesis of DAAT requires acylation, isomerization, and azo-bridging reactions, respectively, with final yields as low as 68% [39]. In view of this, an electrochemical strategy for the one-step synthesis of DAAT is proposed, which can improve atomic utilization while dual-electrode hydrogen production and green synthesis of azo compounds.

The construction of the system cannot be separated from the design of the catalyst, and platinum single atoms/nanoparticles (Pt_s,n_) loaded on three-dimensional conductive carbon cloth using nickel disulfide nanosheets as the carrier were prepared as an efficient HER catalyst (Pt_s,n_@NiS_2_@CC). The Pt_s,n_ can synergistically optimize reaction pathways through electronic interactions (e.g., charge transfer) or spatial effects to enhance overall catalytic activity [40]. For example, clusters may promote reactant adsorption, while single-atom sites accelerate the conversion of key steps [41]. Platinum single-atom catalysts (Pt SAs) are known for their exceptional performance in various reactions, such as photocatalysis [42, 43]. However, their application in alkaline HER is challenging because the reaction involves distinct steps—water dissociation (Volmer) and hydrogen desorption (Heyrovsky/Tafel)—that are difficult for a single type of active site to promote simultaneously. To address this limitation, recent research has focused on catalysts where single atoms and nanoclusters coexist, as their synergy can optimize the entire reaction pathway, showing great promise for complex processes like HER [44, 45]. Notably, single-atom structures are difficult to support complex reactions that require multiple active site synergies and metal–carrier strong interactions (EMSI) may over-stabilize single atoms and lead to performance degradation [46, 47]. Meanwhile, adsorption of reactants or impurities in strongly alkaline environments may lead to active site poisoning [48]. Therefore, Pt_s,n_ has a more stable electronic structure than Pt SA, which makes the composite catalyst more capable of long-term catalysis. In addition, the single-atom performance is limited by the carrier, and the monolayer structure of transition metal sulfide compounds (TMDs) can significantly increase the specific surface area and enhance the loading and dispersion of the active substances compared to the carbon-based ones, thus improving the catalytic or energy storage efficiency [49, 50]. Compared with other TMDs, NiS_2_ flexible structural tunability, dynamic reconfiguration ability, and interfacial synergistic effects are properties that make it superior to conventional TMDs (e.g., MoS_2_, TaS_2_) in terms of active site design, stability enhancement, and wide pH adaptability [51–53]. In addition, the crystal structure of NiS_2_ with the coordination form of Ni^3^⁺ and S_2_^-2^ provides abundant active sites for the material [54–56]. By introducing transition metal (e.g., V, Ru, Pt, etc.) doping, the electronic structure can be further modulated to enhance the catalytic activity [53]. The prepared Pt_s,n_@NiS_2_@CC self-supported electrodes can be directly used in alkaline HER systems, and the DFT theory reveals the synergistic effect between the components. Meanwhile, the DATOR||HER system constructed by combining an anodic catalyst (copper oxide nanowires) realizes dual-electrode hydrogen production and green synthesis of DAAT at ultra-low voltage. The coupled integrated system was stably operated for more than 300 h at industrial-grade current density, which proved the long-term tolerance and stability of the system. The construction of this system provides new ideas and strategies for dual-electrode hydrogen production and green electrosynthesis (Scheme 1b).

## Experimental Section

### Materials

Ammonium persulfate ((NH_4_)_2_S_2_O_8_, AR, ≥ 98%), ammonium fluoride (NH_4_F, AR, ≥ 99%), and urea (CH₄N_2_O, AR, ≥ 99%) were purchased from Tianjin Damao chemical reagent Co., Ltd., China. Carbon cloth (HCP330, thick: 0.36 ± 0.02 mm) was purchased from Shanghai Sanmusk Industrial Co., Ltd., China. Potassium thiocyanate (KSCN, GR, ≥ 99%) and 3,5-diamino-1,2,4-triazole (DAT, AR, ≥ 98%) were purchased from Tianjin Kaida Chemical Reagent Co., Ltd., China. Copper foam (CF, 0.3 mm) was purchased from Ghuangjia Materials Co., Ltd., China. Ruthenium chloride hydrate (RuCl_3_ xH_2_O,35–42.0% Ru basis) was purchased from Shanghai Aladdin Biochemical Technology Co., Ltd., China.

### Preparation of Pt_s,n_@NiS_2_@CC

The NiS_2_@CC composite was synthesized through a controlled thermal sulfidation process. Specifically, 0.6 g of sulfur powder was positioned in the upstream zone of a tubular furnace, while the Ni(OH)_2_@CC precursor was placed in the downstream region. Under argon atmosphere, the system was heated to 300 °C at a ramp rate of 10 °C min^− 1^ and maintained for 2 h to complete the sulfidation reaction. The resultant product underwent thorough purification through multiple CS_2_ washings to remove residual sulfur, followed by drying at 60 °C for subsequent applications. Pt_s,n_ was subsequently deposited on the NiS_2_ surface via cyclic voltammetric electrodeposition (200 CVs) in a three-electrode configuration, employing the prepared NiS_2_@CC as working electrode, a high-purity graphite rod as counterelectrode, and Hg/HgO as reference electrode. The deposition process was conducted within a potential window of -0.6 to 0.2 V (vs. RHE) in 1.0 M KOH electrolyte containing varying concentrations of Pt^4^⁺ solution (10 mg mL^− 1^). Precise control of Pt loading was achieved by adding different volumes (0.1, 0.5, 1.0, 2.0, and 3.0 mL) of Pt^4^⁺ solution, with the resultant catalysts designated as Pt-0.1, Pt-0.5, Pt-1.0, Pt-2.0, and Pt-3.0 accordingly*:*

### Preparation of CuO/CF

The CuO/CF (copper foam, 1 cm × 0.5 cm) was prepared through a two-step process. First, the Cu(OH)_2_/CF precursor was synthesized by reacting the CF in an aqueous solution with a 50 mL NaOH/(NH_4_)_2_S_2_O_8_ molar ratio of 13:1. This reaction was carried out at 25 °C for 40 min without stirring. The precursor was subsequently converted to CuO/CF by annealing in a tube furnace under static air. The annealing procedure involved heating to 300 °C at a ramp rate of 5 °C min^− 1^, maintaining this temperature for 4 h, and then allowing the sample to cool down naturally to room temperature.

## Results and Discussion

### Preparation and Characterization of Cathode Catalyst

**Fig. 1:**
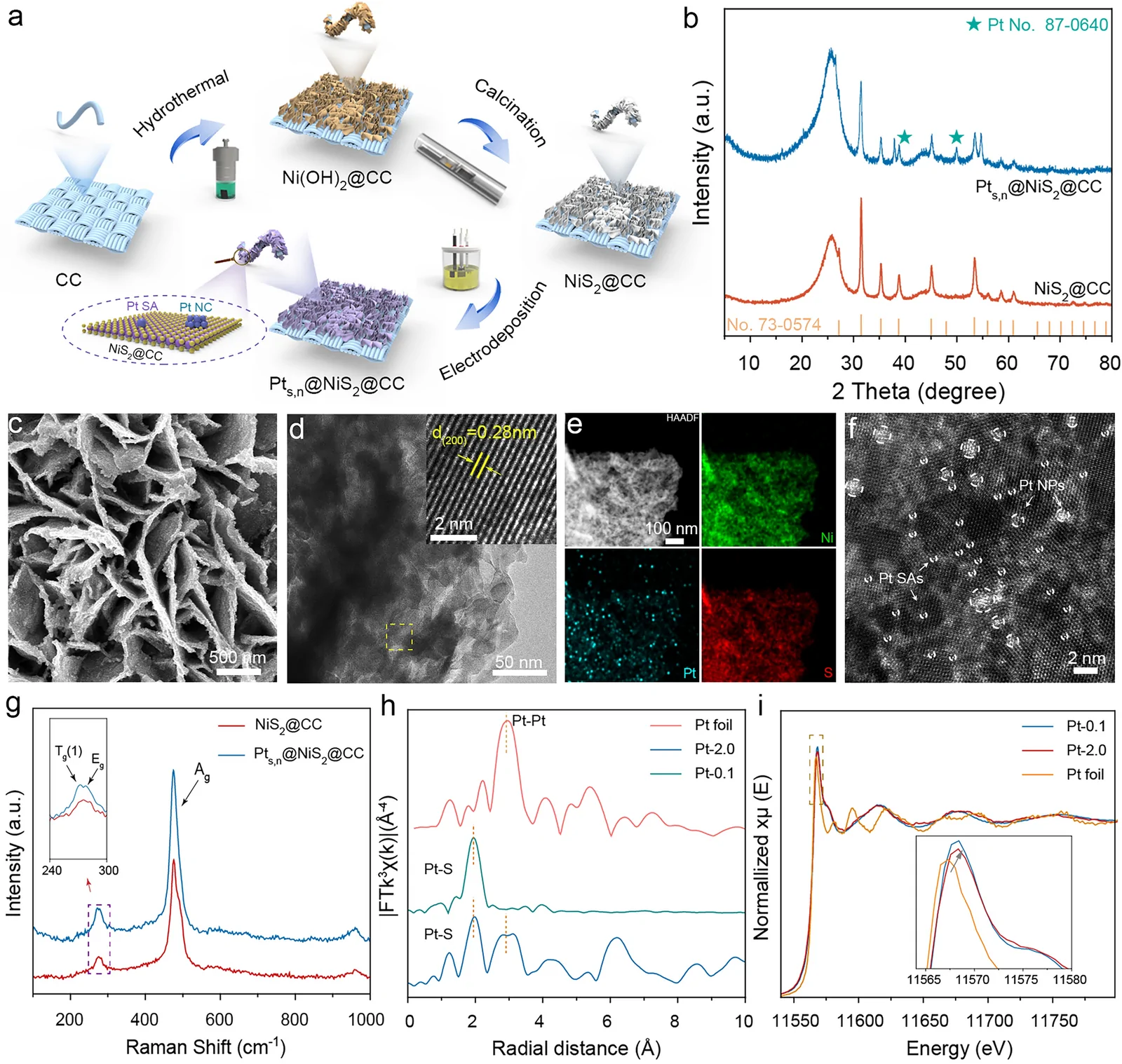
**a** Schematic illustration of the synthesis procedures for Pt_s,n_@NiS_2_@CC. **b** XRD pattern of Pt_s,n_@NiS_2_@CC and NiS_2_@CC. **c** SEM and **d** TEM image of Pt_s,n_@NiS_2_@CC. **e** HAADF-STEM images of Pt_s,n_@NiS_2_@CC. **f** AC-HAADF-STEM images of Pt_s,n_@NiS_2_. **g** Raman spectra of Pt_s,n_@NiS_2_@CC and NiS_2_@CC. **h** K_3_-weighted FT-EXAFS of Pt L_3_-edge for the samples. **i** Normalized Pt L_3_-edge X-ray absorption near-edge structure spectra of Pt foil and samples

The schematic synthesis of carbon cloth (CC)-supported NiS_2_-loaded Pt single atom and Pt cluster (Pt_s,n_) HER catalysts is shown in Fig. 1a. Ni(OH)_2_ with a dense nanosheet structure was first grown on CC by a one-step hydrothermal method. Subsequently, Ni(OH)_2_ grown on CC under Ar atmosphere reacted with S powder at 300 °C to obtain NiS_2_@CC. Finally, Pt_s,n_ was loaded onto the NiS_2_ surface by cyclic voltammetric electrodeposition, resulting in the preparation of a Pt-based composite HER catalyst (Pt_s,n_@NiS_2_@CC). The crystal structure and material composition of Pt_s,n_@NiS_2_@CC were investigated by XRD tests. As shown in Fig. S1a, the precursor Ni(OH)_2_ displays both α and β phases [57, 58]. The α-phase has distinct peaks at 11.4°, 22.7°, and 33.4°, while the β-phase has characteristic peaks at 19.2° and 38.6°. In addition, the diffraction peak at 24.5° is related to the CC substrate. Pt_s,n_@NiS_2_@CC correspond to (200), (220), and (311) crystal planes at 31.4°, 45.0°, and 53.4°, respectively, which can correspond to hexagonal NiS_2_ (PDF No.73–0574). Among them, 40.2° and 46.8° correspond to the diffraction peaks of Pt, which preliminarily proves the successful synthesis of Pt_s,n_@NiS_2_@CC (Fig. 1b). The microstructure and morphology of the samples were characterized using scanning electron microscopy (SEM) and transmission electron microscopy (TEM). Figure S1 shows the nanosheet structure of the precursor, and the 0.23 nm lattice spacing in the corresponding TEM image is attributed to the (011) crystal plane of Ni(OH)_2_. Meanwhile, the corresponding elemental mapping showed a uniform distribution of Ni and O, proving the successful synthesis of pure Ni(OH)_2_. In addition, NiS_2_, obtained after vulcanization, retained the nanosheet structure of the precursor and overlapped with each other to form a three-dimensional interface (Fig. S2). Similarly, the lattice spacing of 0.28 nm is attributed to the (200) crystal plane of NiS_2_, and the elemental mapping corroborates the coexistence of Ni and S elements. The roughness of the surface of Pt_s,n_@NiS_2_@CC is further increased while retaining the nanosheet structure due to the uniform distribution of Pt particles, which increases the specific surface area of NiS_2_ (Fig. 1c). It is worth noting that the 3D nanosheet structure can better adapt to the complex reactions in aqueous phase systems, and a comparison of the contact angles before and after catalyst loading shows that the catalyst loaded on the CC surface possesses good hydrophilicity, which facilitates the solution interface reactions (Fig. S3). The lamellar structure of the samples was further demonstrated by TEM images and the (200) crystalline surface of NiS_2_ was observed, indicating that the electrodeposition process did not affect the material composition and surface structure of NiS_2_ (Figs. 1d and S4). The elemental mapping further indicates the successful loading of Pt with NiS_2_ surface (Fig. 1e). Special aberration-corrected transmission electron microscope (AC-TEM) was further used to further characterize the topographical information of the Pt_s,n_@NiS_2_@CC surface (Fig. 1f).

Among them, Pt nanoparticles (Pt NPs) and Pt single atoms (Pt SAs) coexist, and the lattice spacing of 0.225 nm shows the (111) crystal plane of Pt (Fig. S5). To further investigate the co-presence of Pt atomic sites and nanoparticles in Pt_s,n_@NiS_2_@CC, elemental mapping by AC-HAADF-STEM was performed. As illustrated in Fig. S6, the spatial distribution of Pt on the NiS_2_ nanosheets exhibits both isolated bright spots and agglomerated regions, which are interpreted as corresponding to Pt SA and Pt NPs, respectively. The composite form of Pt_s,n_ provides more types of active sites and avoids the drawbacks due to the inadequacy of a single structure to support complex reactions, thus better facilitating reaction kinetics. The X-ray photoelectron spectroscopy (XPS) survey analysis of Pt_s,n_@NiS_2_@CC confirmed the coexistence of Ni, S, and Pt, as evidenced in the elemental composition profile (Fig. S7a). In order to further reveal the electronic modulation of Pt_s,n_ for NiS_2_ surface, the fine spectra of S, Ni and Pt were analyzed separately (Fig. S7b, c). The Ni 2*p* XPS spectra of Pt_s,n_@NiS_2_@CC revealed two characteristic peaks assigned to Ni^2+^ (853.8 and 871.8 eV) and Ni^3+^ (857.0 and 875.6 eV), with the Ni^3+^ oxidation state attributed to unavoidable surface oxidation. Additionally, the remaining two peaks were identified as satellite features associated with Ni species. These results are consistent with recent reports [59]. The Ni 2*p* XPS spectra of Pt_s,n_@NiS_2_@CC displayed a slight negative shift of 0.4 eV compared to NiS_2_@CC, indicative of charge transfer induced by the incorporation of Pt_s,n_. Similarly, the high-resolution S 2*p* spectrum was resolved into three distinct peaks: The S 2*p*_1/2_ (164.1 eV) and S 2*p*_3/2_ (162.9 eV) components attributed to Ni-S bonds, alongside a SOₓ peak (169.5 eV) associated with Ni–O-S interactions. Notably, a corresponding negative shift of 0.4 eV was observed in the S 2*p* binding energy following Pt_s,n_ modification, consistent with electron transfer from Pt to the NiS_2_. This phenomenon likely arises from direct Pt–S interfacial contact, where the electronegative S atoms withdraw electrons from Pt, forming localized polar covalent or ionic bonds. Such chemical bonding effects further facilitate directional electron delocalization from Pt to NiS_2_, reinforcing the charge redistribution mechanism. More importantly, the electron redistribution induced by Pt_s,n_ can effectively enhance the adsorption of H* by Pt, thus enhancing the HER catalytic activity. The Pt 4f XPS spectra of Pt_s,n_@NiS_2_@CC revealed two characteristic doublets located at 76.2/71.9 and 74.4/71.0 eV, assigned to Pt^δ+^ and Pt^0^ species, respectively [60]. These results further confirm the coexistence of Pt SAs and Pt NPs on the NiS_2_ surface. (Fig. S7d). Raman spectroscopy was used to compare the structural features of the NiS_2_ surface before and after Pt_s,n_ loading. As shown in Fig. 1g, the low-frequency double peaks of pristine NiS_2_ at 278 and 271 cm^− 1^ are attributed to the line pair release mode (T_g(1)_, E_g_ mode), and the high-frequency single peak at 475 cm^−1^ is attributed to the isotropic telescopic vibration of the S–S line pair (A_g_ mode) [61, 62]. The vibrational modes of NiS_2_ were retained after Pt_s,n_ loading, indicating that the introduction of Pt_s,n_ did not destroy the original structure of NiS_2_. In addition, the relative intensity of A^1^_g_/E^2^_1g_ is elevated compared to pristine NiS_2_, suggesting that the loading of Pt_s,n_ acquires more edge defects and rougher surfaces, which are more favorable for the exposure of active sites. X-ray absorption near-edge structure (XANES) analysis was conducted to elucidate the local coordination environment and atomic interactions of Pt_s,n_ on the NiS_2_ surface. In Fig. 1h, dominant peaks at 2.88 and 1.92 Å are assigned to Pt–Pt metallic bonding and Pt–S coordination, respectively. Strikingly, Pt-0.1 exclusively exhibits a primary peak at 1.92 Å, unambiguously confirming the atomic dispersion of Pt as SAs via Pt–S bonding. In contrast, Pt-2.0 displays an additional prominent Pt–Pt peak at 2.88 Å, signifying partial agglomeration of Pt SAs into NPs under higher Pt^4 +^ precursor concentrations, thereby forming a hybrid Pt_s,n_ configuration (single atoms coexisting with nanoparticles). These findings align consistently with prior structural observations (Fig. 1f). Extended X-ray absorption fine structure (EXAFS) spectroscopy further corroborates the coexistence of Pt SAs and Pt NPs. As illustrated in Fig. 1i, the EXAFS profiles of Pt-0.1 (0.1 means that the dosage of Pt^4 +^ is 0.1 mL) and Pt-2.0 exhibit distinct deviations from the reference Pt foil, confirming variations in Pt oxidation states and coordination geometries. Magnified views of the absorption edges reveal that the white line intensities of both Pt-0.1 and Pt-2.0 are positioned above that of Pt foil, indicative of partial positive charges on Pt species. Notably, the higher white line intensity of Pt-0.1 compared to Pt-2.0 suggests a greater proportion of positively charged Pt SAs in the low-concentration sample.

### Electrochemical Properties of Alkaline HER

**Fig. 2:**
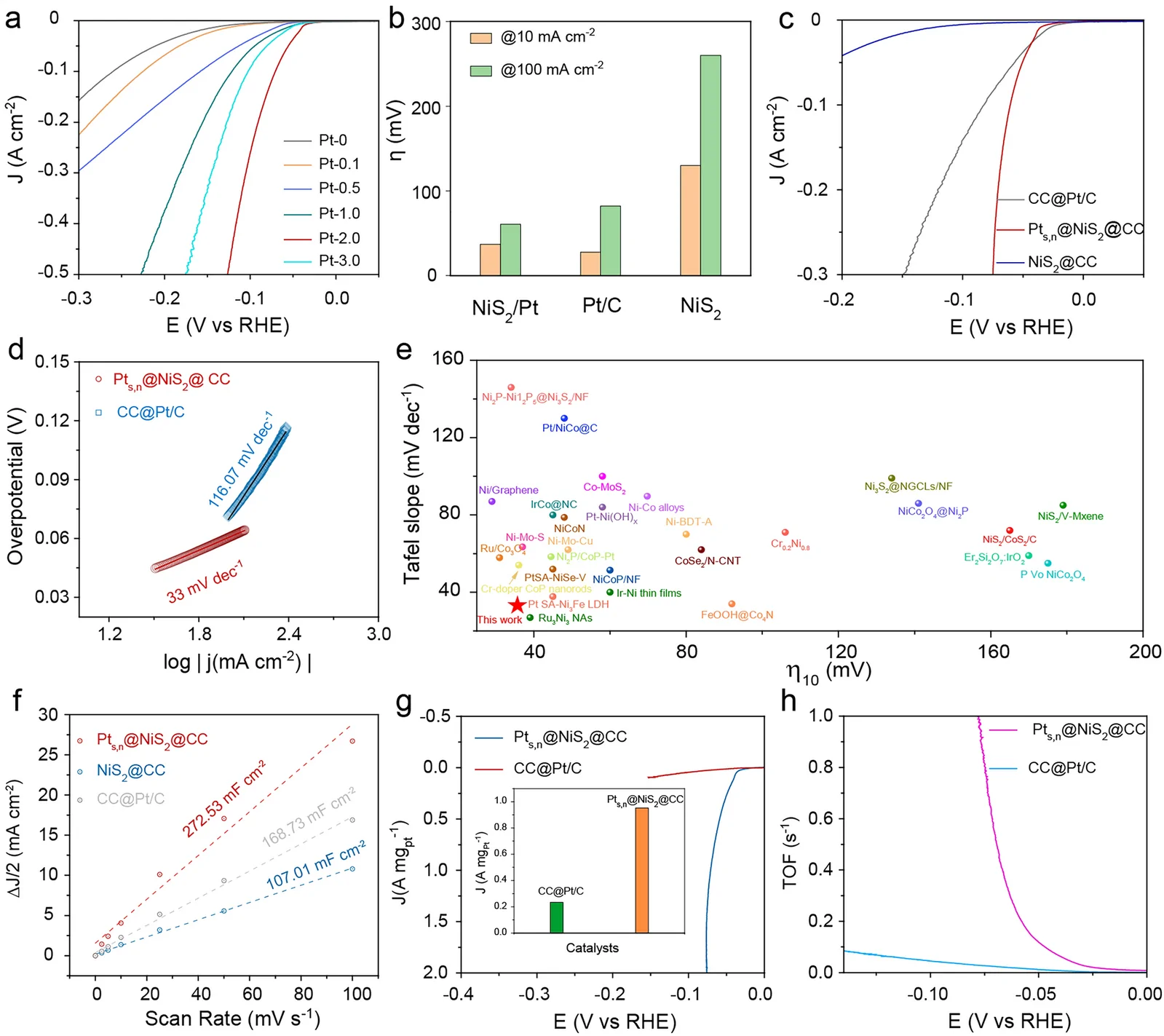
**a** LSV curves of Pt_s,n_@NiS_2_@CC with different Pt^4 +^ contents. **b** Comparison of the overpotential at − 100 mA cm^− 2^ and − 10 mA cm^−2^ for different Pt_s,n_ loadings on NiS_2_@CC. **c** LSV curves of NiS_2_@CC, Pt_s,n_@NiS_2_@CC, and Pt/C. **d** Tafel curve of CC@Pt/C, Pt_s,n_@NiS_2_@CC. **e** comparison of performance of reported HER electrocatalysts at 10 mA cm^− 2^ and Tafel slope. **f** Current density diagrams of NiS_2_@CC, Pt_s,n_@NiS_2_@CC, and Pt/C at different scanning rates. **g** Mass activity curve of Pt_s,n_@NiS_2_@CC and CC@ Pt/C. **h** Turnover Frequency (TOF) plots of Pt_s,n_@NiS_2_@CC and CC@Pt/C

**Fig. 3:**
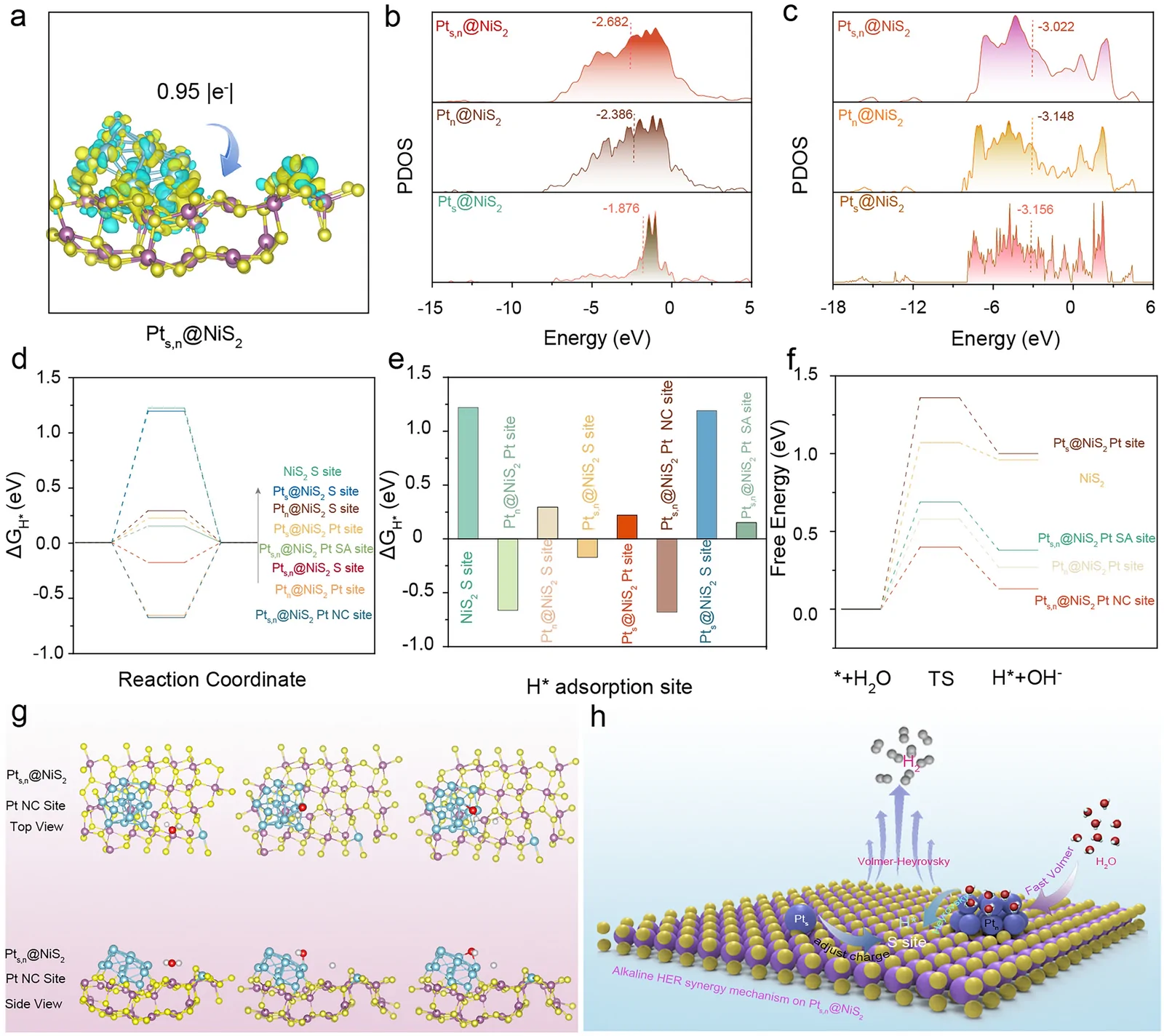
**a** Differential charge density diagram of Pt_s,n_@NiS_2_. The p orbitals density of state of **b** Pt site and **c** S site on the surfaces of Pt_s,n_@NiS_2_, Pt_s_@NiS_2_, and Pt_n_@NiS_2_. **d** Calculated ΔG_H*_ values at various sites of Pt_s,n_@NiS_2_. **e** Comparison of ΔG_H*_ on the S and Pt sites of Pt_s_, Pt_n_, and Pt_s,n_. **f** Free energy of the water dissociation barrier on different sites. **g** Structural models of initial state, transition state, and final state of Volmer pathway on Pt_s,n_@NiS_2_@CC. **h** Illustration of possible synergistically catalytic mechanism of alkaline HER on Pt_s,n_@NiS_2_

To investigate the effect of Pt loading on NiS_2_@CC, the HER properties of the samples were evaluated in 1.0 M KOH. The effect of Pt^4 +^ solution concentration on the HER properties was first evaluated by linear voltammetry (LSV, Fig. 2a). When the Pt solution dosage was 2.0 mL (i.e., Pt-2.0), the sample showed a significant advantage. (60.6 mV@η_100_), and as the Pt dosage continued to increase the actives sites showed a decreasing trend, indicating that there was a peak in the loading of Pt, and the excessive loading occupied the basal sites leading to the attenuation of the HER performance. Furthermore, the catalytic performance of Pt_s,n_ @NiS_2_@CC was evaluated in both 0.5 M and 2.0 M KOH electrolytes. As shown in Fig. S8, the limited OH^−^ availability in 0.5 M KOH slows the Volmer step, while the increased viscosity of 2.0 M KOH impedes mass transport. Therefore, 1.0 M KOH is identified as the optimal electrolyte concentration for the hydrogen evolution reaction. The decay of Pt-3.0 HER activity is affected by both a decrease in the number of active sites and changes in the electronic structure of the surface (e.g., H* adsorption strength). Firstly, TEM images of Pt-3.0 reveal that the average size of Pt NPs increases significantly from about 2.93 nm (Pt-2.0) to approximately 4.60 nm, accompanied by noticeable agglomeration. Furthermore, XPS analysis shows that the peak intensity at 74.4 eV for Pt-3.0 increases. Compared to Pt-2.0, indicating that among the detectable surface Pt species, Pt in Pt-3.0 retains more of its metallic nature, with an electronic structure closer to neutral Pt^0^ and a significantly higher proportion of metallic electronic characteristics (Fig. S9). Therefore, the formation of Pt particles reduces the number of active sites, leading to decreased HER activity. On the other hand, Pt particles exhibit relatively high H* adsorption capacity, preventing smooth desorption of H* during the Volmer step and resulting in the decay of HER activity for Pt-3.0 (see Fig. 3 for details). The experimental results demonstrate distinct inhibitory effects of different chemical agents on platinum species: EDTA primarily inhibits Pt Pt SAs), while thiocyanate ions (SCN⁻) exhibit detrimental effects on both Pt SAs and Pt Pt NPs. As evidenced in Fig. S10, the introduction of EDTA induced a moderate decrease in the HER performance of Pt_s,n_@NiS_2_@CC, while subsequent addition of KSCN in the electrolyte triggered more pronounced activity degradation, confirming the predominant role of Pt. Figure 1h results show that Pt-0.1 surface is mainly dispersed in the form of Pt SAs and Pt-2.0 forms a hybrid structure consisting of individual atoms and nanoparticles. Given this structural difference, the hydrogen evolution reaction (HER) performance of Pt-0.1 and Pt-2.0 was evaluated in 1.0 M KOH electrolyte before and after the addition of 10 mM EDTA. As shown in Fig. S11, the overpotential of Pt-0.1 increased significantly by 237 mV after EDTA introduction, whereas Pt-2.0 exhibited a smaller performance degradation, with an overpotential increase of only 148 mV. This discrepancy can be attributed to the extensive coordination of Pt single atoms in Pt-0.1 with EDTA, leading to the deactivation of active sites. In Pt-2.0, however, the presence of Pt nanoparticles alongside single atoms helps mitigate the performance loss caused by such coordination. According to XANES, it is known that Pt exists as Pt SA in Pt-0.1. However, the η_100_ of Pt-0.1 is 224.9 mV, which is significantly higher than that of Pt-2.0 (i.e., Pt_s,n_@NiS_2_@CC), further suggesting that a single loading form is not sufficient for a better HER performance. In addition, Pt_s,n_@NiS_2_@CC also shows a lower overpotential (36.9 mV) at a current density of 10 mA cm^− 2^, which is slightly higher than that of commercial Pt/C (27.8 mV). However, at higher current densities, Pt_s,n_@NiS_2_@CC showed a tendency to outperform the commercial Pt/C (Fig. 2b, c).

In order to systematically evaluate the effect of different transition metal sulfides as carriers on the performance of hydrogenation reaction (HER), MoS_2_- and VS_2_-loaded materials were prepared on carbon cloth (CC) substrate, and Pt monoatomic and nanoparticle (Pt_s,n_) active centers were constructed on the surfaces of them by the same Pt deposition process, respectively. Pt_s,n_@MoS₂@CC and Pt_s,n_@VS_2_@CC composite electrodes were successfully synthesized. Linear scanning voltammetry (LSV) test results showed that Pt_s,n_@NiS_2_@CC exhibited optimal HER electrocatalytic activity under the same test conditions. When the current density reached 100 mA cm^−^^2^, the required overpotential of Pt_s,n_@NiS_2_@CC was only 60.6 mV, which was significantly lower than that of Pt_s,n_@MoS_2_@CC (80.2 mV) and Pt_s,n_@VS_2_@CC (81.3 mV). The above results indicate that the Pt_s,n_ catalyst with NiS₂ as the carrier has more prominent advantages in catalytic performance in HER (Fig. S12). The HER mechanism on the Pt_s,n_@NiS_2_@CC electrocatalyst was investigated through Tafel slope analysis (Fig. 2d). The Pt_s,n_@NiS_2_@CC exhibited a significantly lower Tafel slope of 33.0 mV dec^−1^ compared to 116.07 mV dec^− 1^ for Pt/C, confirming the dominance of a rapid Volmer–Heyrovsky pathway and accelerated HER kinetics on its surface. Furthermore, the overpotential at 10 mA cm^− 2^ and Tafel slope of Pt_s,n_@NiS_2_@CC in 1.0 M KOH were benchmarked against state-of-the-art HER catalysts (Fig. 2e and Table S1). The results demonstrate that Pt_s,n_@NiS_2_@CC outperforms most reported electrocatalysts in alkaline media, highlighting its superior HER activity and efficient mass/charge transport dynamics [63]. In addition, the electron transport dynamics on the Pt_s,n_@NiS_2_@CC surface was investigated by electrochemical impedance spectroscopy (EIS). Figure S13 reveals distinct charge transfer resistances (R_ct_) for CC@Pt/C, NiS_2_@CC, and Pt_s,n_@NiS_2_@CC, with values of 25.0, 2.26, and 0.78 Ω cm^2^, respectively. The markedly lower R_ct_ of Pt_s,n_@NiS_2_@CC underscores its enhanced electron transport kinetics during the HER process, attributable to the synergistic interplay between Pt single atoms/nanoparticles (Pt_s,n_) and the conductive NiS_2_@CC substrate. The electrochemically active surface area (ECSA) is proportional to the capacitance of the bilayer (C_dl_) and represents the actual electrochemical active surface area. Figure S14 shows the cyclic voltammetry curves corresponding to the samples at different scanning speeds and fitted to obtain C_dl_ values. As shown in Fig. 2f, compared with CC@Pt/C (168.73 mF cm^− 2^) and NiS_2_@CC (107.01 mF cm^− 2^), Pt_s,n_@NiS_2_@CC (272.53 mF cm^− 2^) has a higher C_dl_, indicating that it has a larger ECSA and more exposed active sites. In addition, the data show that after ECSA normalization, commercial Pt/C indeed exhibits mass-specific activity comparable to or slightly higher than Pt_s,n_@NiS_2_@CC in the low overpotential region (Fig. S15). Inductively Coupled Plasma Optical Emission Spectrometer (ICP-OES) measured that the loading of Pt_s,n_ on the surface of NiS_2_@CC was 0.163 mg cm^− 2^. Based on this result, the mass activity (MA) of the sample was calculated to reflect the intrinsic activity and atomic utilization of the sample (Fig. 2g). Pt_s,n_@NiS_2_@CC exhibited an MA value of 0.952 A mg^−1^ at 80 mV overpotential, which was approximately 4.06 times higher than that of commercial Pt/C electrodes. In order to evaluate the intrinsic activity of the samples, the turnover frequencies (TOF) of Pt_s,n_@NiS_2_@CC and Pt/C, defined as the number of molecules (H_2_) produced per second per site, were calculated. As shown in Fig. 2h, the TOF value of Pt_s,n_@NiS_2_@CC is higher than that of Pt/C in a wide range of overpotentials, reaching 0.568 s^−1^ at 70 mV as 23.9 times that of commercial Pt/C electrodes. Stability, a pivotal criterion for assessing the long-term viability of electrocatalysts, was systematically evaluated for Pt_s,n_@NiS_2_@CC. Figure S16a compares the LSV profiles of CC@Pt/C and Pt_s,n_@NiS_2_@CC before and after 2000 cyclic voltammetry (CV) cycles. Unlike CC@Pt/C, which exhibits significant activity degradation, Pt_s,n_@NiS_2_@CC retains highly overlapping LSV curves with negligible polarization shift, demonstrating exceptional structural robustness and operational durability. Furthermore, chronopotentiometry (CP) tests were conducted to validate the stability under continuous HER operation (Fig. S16b). Remarkably, Pt_s,n_@NiS_2_@CC maintains stable catalytic performance with < 2% potential decay over 100 h at a current density of 10 mA cm^−2^, in stark contrast with the substantial voltage oscillations observed for CC@Pt/C. This minimal performance attenuation underscores the superior interfacial stability and anti-agglomeration capability of the Pt_s,n_-modified structure, which effectively mitigates Pt leaching or coalescence during prolonged electrocatalysis. In addition, to evaluate the structural and chemical stability of the material, we performed systematic characterization of the sample after CP testing. The XRD pattern shows that all diffraction peaks remain well matched with the standard NiS_2_ phase (JCPDS No.73–0574), indicating that the crystal structure is retained after testing. The XPS survey spectrum confirms the coexistence of Ni, S, and Pt in the sample. Moreover, high-resolution XPS spectra reveal that the binding energies of each element remain largely unchanged compared to their pre-reaction states, demonstrating that Pt_s,n_@NiS_2_@CC maintains good chemical stability during the long-term test. Raman spectroscopy further supports the structural integrity of the sample, as the characteristic vibration modes of Pt_s,n_@NiS_2_@CC are preserved after testing. SEM images show that the flake-like morphology remains intact, and TEM analysis confirms that Pt is uniformly distributed on the NiS_2_ surface. Collectively, these results provide strong evidence that Pt_s,n_@NiS_2_@CC exhibits excellent structural and chemical stability under harsh reaction conditions (Fig. S17). Subsequently, the Faraday electron efficiency (FE) of Pt_s,n_@NiS_2_@CC was measured using the drained gas collection method. Hydrogen started to be produced when a constant current density was applied to the system, as shown in Fig. S18, and hydrogen was quantified every 20 min, which showed a positive relationship between hydrogen production and time. Meanwhile, the calculated FE of Pt_s,n_@NiS_2_@CC is close to 100% indicating the ability of Pt_s,n_@NiS_2_@CC to produce hydrogen in a stable manner.

### Mechanistic Investigation of Alkaline HER

Density functional theory (DFT) calculations were performed to unravel the intrinsic interplay and synergistic effects among Pt SAs, Pt NPs, and the NiS_2_ substrate. Three distinct computational models Pt_s_/NiS_2_, Pt_n_/NiS_2_, and Pt_s,n_/NiS_2_ were constructed with full geometric relaxation (Fig. S19). Given the computational infeasibility of modeling Pt nanoparticles (average size: 2.93 nm) with DFT, we employed a Pt_13_ cluster, for which the lowest energy structure was determined using density functional theory (DFT), as a simplified model [44, 45]. Charge density difference analysis (Fig. 3a) revealed pronounced electron redistribution at the Pt_s,n_@NiS_2_ interface, where electrons were transferred from Pt species to the NiS_2_ substrate. This interfacial charge transfer induces polarization of Ni-S bonds, effectively lowering the hydrogen adsorption energy (ΔG_H*_) and generating highly active HER sites at the heterojunction. Notably, the depletion of electron density at Pt sites and its accumulation on adjacent NiS_2_ regions corroborate the Pt to NiS_2_ electron donation mechanism observed in XPS studies. Comparative quantification of charge transfer magnitudes revealed that Pt_s,n_/NiS_2_ exhibited the highest electron transfer (ΔQ = 0.95 e^−^), significantly surpassing Pt_s_/NiS_2_ (ΔQ = 0.20 e^−^) and Pt_n_/NiS_2_ (ΔQ = 0.69 e^−^) (Figs. 3a and S20). This enhanced charge delocalization in the Pt_s,n_ system facilitates robust electronic metal–support interactions (EMSI), which stabilize Pt species and optimize interfacial reactivity. Figure S21 shows that the S, Ni and Pt orbitals of the Pt_s,n_/NiS_2_ model have a high degree of electronic state overlap, further indicating an effective interaction between Pt_s,n_ and the interface. Based on this, van der Waals forces and weak covalent interactions between Pt_s,n_ and the interface are hypothesized [64]. P-band center (ε_p_) is used as a descriptor, which can significantly affect the adsorption strength of H* by regulating the electronic structure on the surface of the material and then evaluate the activity of the catalyst. Optimizing the position of ε_p_ (such as doping and defect design) is an effective strategy to improve the efficiency of catalytic reactions such as HER. Therefore, the P orbital of S and the D orbital of Pt are calculated to explain the different adsorption capacities of H* or H_2_O on the three active sites. As shown in Fig. 3b, compared with Pt_s_/NiS_2_ and Pt_n_/NiS_2_, the Pt_s,n_/NiS_2_ configuration exhibits a upshifted ε_p_ at the S sites, in which, according to the p-band center theory, a higher ε_p_ usually corresponds to a stronger hydrogen adsorption capacity [65, 66]. This electronic structure modulation is a crucial component of the overall synergistic effect. Similarly, at the Pt site, Pt_s,n_@NiS_2_ has a more negative ε_p_, which shows the weak adsorption of Pt site (Fig. 3c). These results confirmed that the interaction between NiS_2_ and Pt effectively accelerated the electron transfer, reduced the catalytic energy barrier, and improved the catalytic activity of the catalyst. ΔG_H*_ (adsorption of H*) is considered as the most ideal HER catalyst when it approaches zero, that is, the balance between the reduction rate of protons and the removal rate of H*. H* adsorption models were constructed for different loci, and ΔG_H*_ was calculated for the relevant loci (Figs. 3d and S22). Figure 3e records the values of ΔG_H*_ for different sites. The composite Pt_s,n_@NiS_2_ (− 0.18 eV) shows a stronger thermal center compared to Pt_s_@NiS_2_ (1.19 eV) and Pt_n_@NiS_2_ (0.29 eV), which suggests that the Pt_s,n_ composite structure contributes to the regulation of the electrons and thus shows a more suitable H* intensity. Notably, the Pt_s,n_@NiS_2_ S site showed a more moderate ΔG_H*_ than the Pt site, suggesting that the S site of NiS_2_ may be an active center for H adsorption/desorption, thus promoting the Heyrovsky process. However, the process of hydrolysis dissociation (Volmer) is usually considered the decisive step in alkaline water electrolysis. Different water adsorption models were constructed and model optimized for their transition states to explore the mechanism of the role of Pt_s,n_ in the Volmer process (Figs. 3g andS23, S24, S25 and S26). As shown in Fig. 3f, which shows the free energy diagram of the Volmer step of Pt_s,n_@NiS_2_, it can be seen that the Pt_n_ site has a lower reaction barrier than that of Pt_s_, suggesting that the introduction of Pt_n_ accelerates the Volmer step and thus promotes the production of more H*, which provides sufficient substrate for the subsequent Heyrovsky step. Based on the above discussion, a synergistic reaction mechanism of Pt_s,n_ and NiS_2_ is proposed. As shown in Fig. 3h the introduction of Pt_n_ greatly reduced the potential barrier for H_2_O dissociation, making H* extremely easy to produce. Meanwhile, Pt_s_, as an effective electron modulator, improved the surface charge distribution of NiS_2_ and polarized the Ni-S bond, which made the S site more thermally neutral, thus promoting the H* adsorption process. (i.e., Volmer step). As a result, the Pt_s,n_@NiS_2_ surface forms a unique Volmer–Heyrovsky pathway, which ensures the stable adsorption of H* and avoids its over-strong adsorption leading to desorption difficulties, thus enhancing the overall reaction kinetic efficiency.

### Anode DATOR Electrochemical Properties and Pathway

**Fig. 4:**
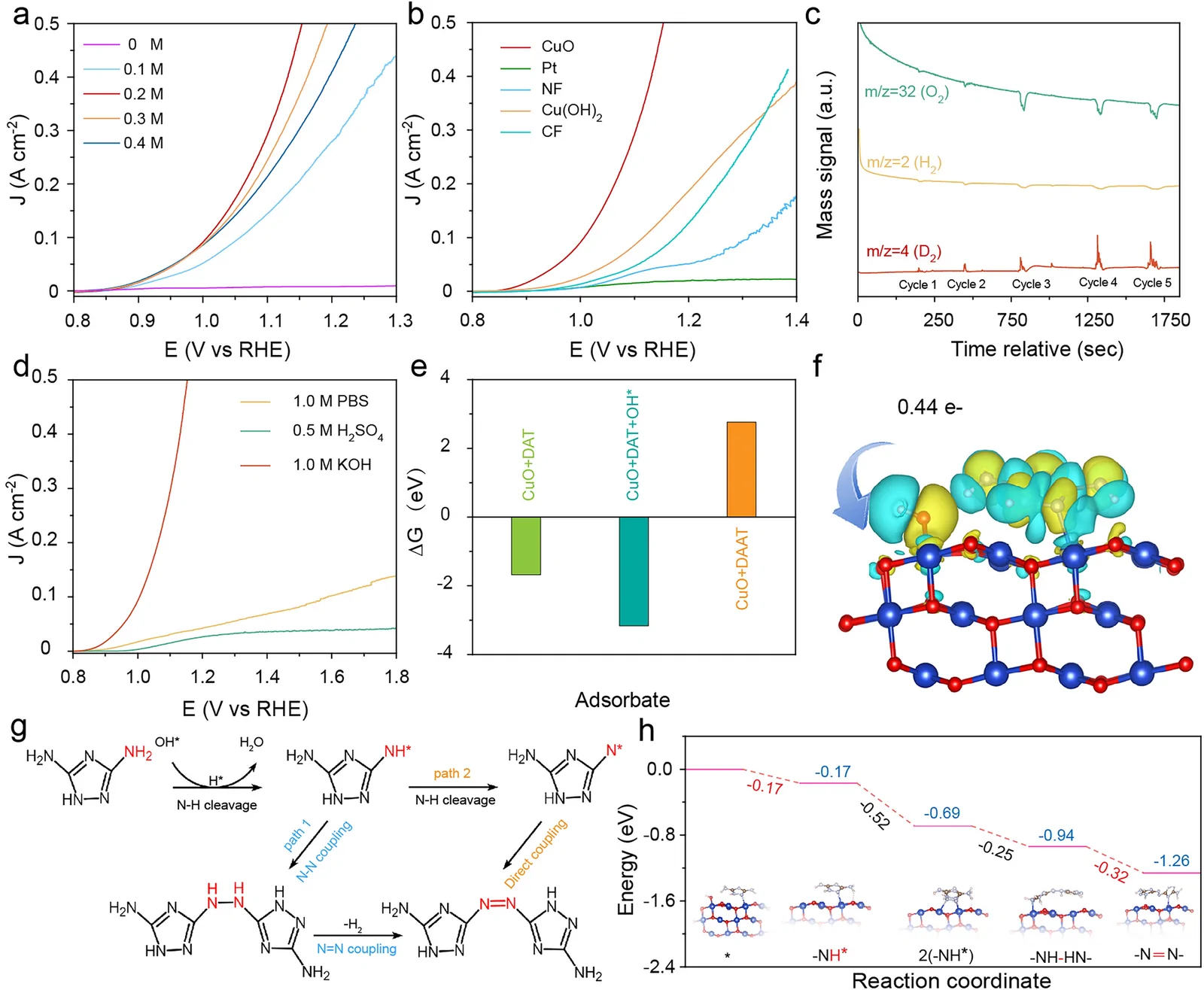
**a** DATOR performance curves of different concentrations of DAT on CuO/CF. **b** DATOR performance curves of different catalysts. **c** In situ DEMS tests of the deuterated DATOR for five LSV cycles. **d** DATOR performance curves of different pH. **e** Comparison of ΔG_H*_ on CuO surface of DAT, DAT + OH^*^ and DAAT. **f** Differential charge density diagram of DAT + OH^*^ on CuO surface. **g** Mechanism diagram of OH^*^ induced DATOR. **h** Relative energy of DATOR pathway (path 1)

The four-electron transfer process inherent in the OER imposes significant thermodynamic barriers, necessitating high energy input for conventional water electrolysis. To mitigate this inefficiency, replacing OER with thermodynamically favorable organic oxidation reactions, characterized by lower oxidation potentials, offers a dual benefit: substantial energy savings and co-production of high-value-added organic chemicals alongside hydrogen. However, the market demand disparity between H_2_ (mass-scale commodity) and organic anode products (niche chemicals) inevitably leads to anode product overproduction, creating a critical scalability challenge. The dual-electrode hydrogen production strategy emerges as an innovative solution to address this market size mismatch. By integrating symmetrical or asymmetric electrocatalytic systems capable of simultaneously generating H_2_ at both electrodes, this approach not only eliminates anode overcapacity but also doubles the H_2_ yield per unit energy input, thereby enhancing process economics and scalability. Such systems leverage pH-gradient control, redox mediator engineering, or dual-functional catalysts to decouple H_2_ production from stoichiometric constraints, enabling flexible adaptation to market-driven H_2_ demand. In view of this, the oxidation reaction of 3,5 diamino-1,2,4-triazole (DATOR) is utilized to replace the OER, which effectively reduces the water decomposition voltage while greenly preparing DAAT energy-containing materials. More importantly, the DATOR process is accompanied by the output of H_2_, which realizes anode hydrogen production and makes up for the shortcoming of insufficient H_2_ capacity. According to previous studies, CuO has an efficient electrochemical azotization ability, as well as an inhibitory effect on OER. Therefore, copper foam (CF)-loaded copper oxide nanowires (CuO/CF) were employed as a catalyst for DATOR. The schematic preparation of CuO/CF is shown in Fig. S27. CF was chemically oxidized in situ and calcined in a process to obtain CuO/CF with a nanowire-like structure. The SEM image of the precursor Cu(OH)_2_ is shown in Fig. S28, with dense nanowire-like structures uniformly attached to the CF surface. Notably, the linear structure has good hydrophilicity, which improves the mass transfer at the solution interface (Fig. S29). XRD images corroborated the successful preparation of CuO/CF, while the morphological features of Cu(OH)_2_ and CuO were observed by SEM and TEM. Figure S30 shows that CuO/CF retained the morphological features of precursor nanowires (NWs) with a lattice spacing of 0.25 nm corresponding to the (111) crystallographic plane of CuO. In addition, elemental mapping shows a uniform loading of Cu and O elements, all evidence of the successful preparation of CuO NWs. The effect of concentration on the performance of DATOR was explored by LSV curves, and it was concluded that DATOR had the best performance when 0.2 M DAT was added (Fig. 4a). Importantly, when DAT was not added (i.e., OER), almost no current was generated under the potential window of 0.8 ~ 1.3 V, indicating the poor OER catalytic ability of CuO/CF, which is more favorable for DATOR. The DATOR performance was further evaluated in 0.5 M and 2.0 M KOH electrolytes. In 0.5 M KOH, the low OH⁻ concentration limits the formation of the OH* intermediate, leading to kinetically sluggish DATOR behavior. In contrast, while 2.0 M KOH provides ample OH^−^, the reduced solubility of DAT in the highly concentrated electrolyte results in a slight performance decline. Therefore, 1.0 M KOH offers the optimal balance, maximizing both intermediate generation and reactant availability for enhanced DATOR activity (Fig. S31). Compared to other catalysts, CuO/CF has a lower start-up voltage, requiring only 1.01 V power input to achieve a current density of 100 mA cm^− 2^, supporting the efficient DATOR activity of CuO/CF (Fig. 4b). In situ differential electrochemical mass spectrometry (DEMS) was utilized to validate the mechanism of H_2_ production at the anode. Figure 4a shows the gap between the reaction potentials of OER and DATOR. Based on this discrepancy, the potential interval of the DEMS test was set to be 0.7–1.6 V and several cycles were performed to explore the source of the anodic H_2_ acquisition. 0.2 M DAT was completely immersed into the deuterium oxide, so that the active H in DAT was fully exchanged with the deuterated reagent, and the fully deuterated DAT substrate was obtained. DEMS recorded information on the mass-to-charge ratio (m/z) of the gas during multiple cycles and detected a source of m/z = 4 (D_2_), the generation of which proved that the anodic H_2_ production originated from the Tafel process after DAT dehydrogenation (Fig. 4c). In addition, gas signals of m/z = 2 (H_2_), m/z = 3 (HD) and m/z = 32 (O_2_) were not detected, a phenomenon that demonstrates that no OER reaction is involved at potentials of at least 1.6 V. Since the only source of H species during the DEMS test was the Heyrovsky process, the non-detection of HD and H_2_ proves that all anodic hydrogen production comes from the de-H process of DAT.

Given that the DATOR occurs in alkaline media, the influence of adsorbed hydroxyl radicals (OH*) on its performance cannot be neglected. To investigate this phenomenon, 2,2,6,6-tetramethylpiperidinyl-1-oxide (TEMPO) and isopropanol (IPA) were employed as hydroxyl radical (OH*) scavengers in the electrochemical system, effectively suppressing the concentration of free OH* radicals within the electrolyte through selective quenching reactions. As demonstrated in Fig. S32, the introduction of TEMPO and IPA led to significant activity decay in DATOR compared to the pristine system, providing preliminary evidence for the OH* critical role in facilitating the reaction. To further validate this hypothesis, we examined DATOR performance under controlled pH conditions (acidic, neutral, and alkaline). Consistent with expectations, the original alkaline environment (pH = 14) exhibited markedly superior catalytic activity relative to acidic and neutral conditions (Fig. 4d), reinforcing that higher OH* availability enhances DATOR kinetics. These findings collectively confirm that OH^*^ act as key mediators in the DATOR mechanism, where their abundance under alkaline conditions promotes efficient reaction progression. In order to verify this concept, the influence of OH^*^ on its reaction path was investigated by DFT calculation. Firstly, a model for adsorbing DAT and DAAT on CuO was constructed, and the resulting model was completely relaxed (Fig. S33). Figure 4e, respectively, shows the adsorption energy values of DAT, DAAT and (DAT + OH*) on the CuO surface. Notably, DAT can show a more negative ΔG (-3.15 eV) with the involvement of OH*, suggesting that OH* can enhance the adsorption of DAT on the surface, which makes it easier to reach the catalyst surface for electrochemical reactions. Differently, DAAT has a more positive ΔG on the CuO surface, showing a weaker adsorption, thus facilitating the stripping of the product from the catalyst surface and avoiding the occupation of the surface active sites due to the strong adsorption, which affects the continuity of the electrochemical reaction. The influence of DAT + OH^*^ on the local electrons of CuO is analyzed by differential charge density diagram (Yellow and cyan represent the aggregation and absence of electrons, respectively). Figures 4f and S34 show the charge distribution of DAT on the surface of CuO, and it can be seen that the electrons of DAT are clustered on the surface of CuO, and this clustered state of electrons strengthens the intramolecular bonding cooperation, which is detrimental to the reaction. In contrast, the introduction of OH* induced the charge off-domain on the surface of DAT, which resulted in weakened intramolecular bonding cooperation and thus more favorable for N–H bond breaking. Baber charge further demonstrated that OH* could enable more electron transfer to the catalyst surface, which in turn increased the electron transport capacity of the catalyst surface and improved the electrochemical polarization phenomenon.

To elucidate the mechanistic role of OH* as a reaction initiator in the DATOR, density functional theory (DFT) calculations were employed to determine the minimum energy pathway (MEP) for DAT decomposition on the catalyst surface (Fig. S35). In the absence of OH*-mediated activation (Paths 3 and 4), the dehydrogenation of the -NH group in DAT exhibited an exceptionally high energy barrier of 0.43 eV, identified as the rate-determining step (RDS) for the entire decomposition pathway (Fig. S36). This substantial energy requirement would severely impede DAT activation and subsequent reaction progression. Notably, the introduction of OH* (paths 1 and 2) dramatically reduced the energy barrier to − 0.17 eV, providing direct evidence for OH* acting as a kinetic promoter to initiate DATOR (Fig. 4g, h). Furthermore, comparative analysis of competing pathways revealed that the formation of -NH-NH- intermediates via -NH coupling (Path 1) exhibited a lower reaction barrier compared to the direct -N = N- bond formation pathway. This thermodynamic preference confirms that Path 1 dominates the DATOR mechanism due to its minimized energy landscape. Based on the mechanistic investigation, a distinct reaction pathway was elucidated for the DATOR process. As demonstrated in Fig. S37, the initial step involves OH-mediated cleavage of the N–H bond in the DAT molecule, resulting in the generation of reactive -NH intermediates. These activated species subsequently undergo spontaneous coupling through intermolecular N–N bond formation. The reaction pathway further progresses via dehydrogenation processes accompanied by concurrent formation of an azo (–N = N–)-containing structure, ultimately yielding molecular hydrogen (H_2_) and the dimerized product DAAT as final outputs. This proposed mechanism highlights the synergistic roles of radical-mediated activation and controlled dehydrogenation in governing the transformation pathway.

### Electrochemical Performance Test of Coupling System

**Fig. 5:**
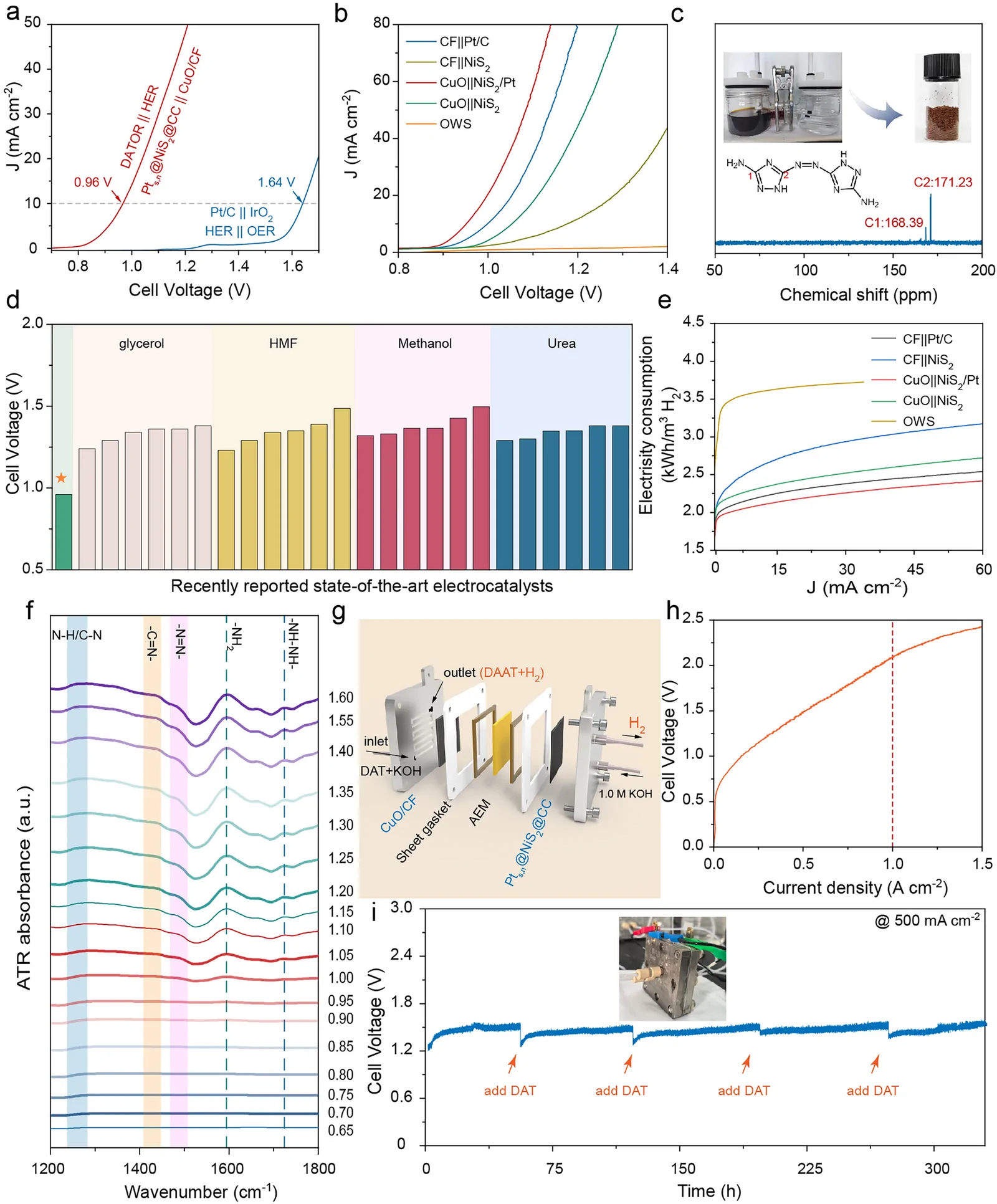
**a** DATOR and OWS performance curve of H-type electrolytic cell. **b** DATOR performance curve of H-type electrolytic cell with different catalysts. **c** ^13^C NMR spectrum of DAAT products. Inset: physical drawing of DAAT. **d** Comparison diagram of cell voltage of different coupling systems. **e** Analysis of consumed electricity of different coupling systems. **f** In situ ATR-FTIR spectra of DATOR over the specified potential ranges. **g** Schematic illustration of an AEMWE. **h** LSV curves of AEMWE. i Long-term stability test of the AEMWE electrolyzer at 500 mA cm^−2^.

Benefiting from the superior electrochemical performance of DATOR and HER, a hybrid water electrolysis coupling system (DATOR||HER) was successfully constructed. In coupling system, the as-prepared CuO/CF composite was employed as the anode to catalyze the DATOR process, while Pt_s,n_@NiS_2_@CC served as an efficient cathode catalyst for the HER, thereby achieving sustainable hydrogen production (Fig. S38). As illustrated in Fig. 5a, the DATOR||HER system assembled in an H-type electrolytic cell requires a cell voltage of only 0.96 V to achieve a current density of 10 mA cm^− 2^. Notably, this represents 680 mV reduction in operational voltage compared to the conventional OER||HER (overall water splitting, OWS, Pt/C||IrO_2_) configuration under identical conditions, where the latter exhibits a significantly higher cell voltage of 1.64 V. This remarkable voltage difference conclusively demonstrates the effectiveness of DATOR in replacing OER for substantially lowering energy consumption in hydrogen production systems. Furthermore, to investigate the stable catalytic performance of CuO/CF, long-term electrolysis tests were conducted at a current density of 500 mA cm^–2^ using the CP test. The XRD pattern confirms that the crystalline phase of the material remains as CuO, with no notable reduction to other copper oxides or metallic copper. The XPS survey spectrum verifies the coexistence of copper and oxygen. In the high-resolution Cu 2*p* spectrum, the principal peaks located at binding energies of 933.75 and 953.73 eV are assigned to Cu 2*p*_3/2_ and Cu 2*p*_1/2_, respectively. Moreover, the presence of distinct satellite peaks at 943 and 962 eV further supports the existence of Cu^2+^ species in the sample. The SEM images reveal that the CuO nanowire morphology is well maintained, showing no evident collapse or detachment from the CF substrate. The TEM observations further indicate that the nanowire structure of CuO is preserved, exhibiting a lattice spacing of 0.25 nm corresponding to the (111) plane of CuO. The elemental mapping further confirmed the coexistence of Cu and O elements. These results provide strong evidence for the exceptional morphological and structural stability of the CuO/CF anode under industrial-grade operating conditions, which is instrumental in ensuring its long-term electrochemical performance (Fig. S39). Figure 5b comparatively evaluates the electrochemical performance of DATOR||HER systems employing different catalytic configurations. Significantly, the DATOR||HER system incorporating DAT exhibited superior overall performance to conventional overall water splitting (OWS), with the CuO/CF||Pt_s,n_@NiS_2_@CC configuration maintaining outstanding catalytic activity throughout the evaluation. DAAT samples were prepared by prolonged CP tests to confirm its material composition. As shown in Fig. 5c, the reacted solution showed a dark brown color, and an orange solid was obtained after acidification, thermal filtration, and lyophilization operations. ^13^C NMR spectra showed two carbon environments at the chemical shifts δ = 168.39 and 171.23 ppm attributed to C-NH_2_ and C = N species, respectively. This is consistent with previous reports, which proves the successful synthesis of DAAT [67]. It is worth noting that the DATOR||HER coupling system with ultra-low cell voltage shows lower energy consumption than the recent new coupling systems (glycerol, 5-hydroxymethylfurfural (HMF), methanol, and urea) (Fig. 5d and Table S2). Due to the low oxidation potential of DATOR, the coupling system only needs the power consumption of 2.38 kWh m^− 3^ H_2_ at the current density of 30 mA cm^− 2^, which saves 35.8% of the power input compared with the traditional OWS (3.71 kWh m^− 3^ H_2_@30 mA cm^− 2^, Fig. 5e). The formation process of DAAT was observed by in situ ATR-FTIR spectroscopy. It has been proved that DATOR has no obvious OER reaction within at least 1.6 V (Fig. 4c). Therefore, the ATR-FTIR spectral information at different voltages was recorded by applying different potentials (0.65–1.6 V) under uncompetitive OER conditions (Fig. 5f). As revealed by spectroscopic analysis, no discernible spectral signatures were detected below 0.9 V (vs RHE), indicating kinetically restricted DATOR activation under these low-potential conditions. Upon progressive voltage elevation, characteristic vibrational bands at 1280, 1435, and 1630 cm^−1^ exhibited intensity enhancements corresponding to N–H/C-H stretching, C = N vibration, and NH_2_ deformation modes of DAT molecules, respectively. This spectral evolution demonstrates potential-dependent adsorption dynamics, where increased DAT coverage on the catalytic surface (CuO/CF) promotes charge transfer efficiency, thereby enabling sustained reaction progression at elevated potentials. More importantly, the emergence of OH* species (~ 3600 cm^− 1^) and -NH-NH- moieties serves as direct spectroscopic evidence for OH^*^-mediated N–N bond coupling during DATOR initiation (Fig. S40). This observation aligns with computational simulations of Path 1 in the proposed reaction mechanism (Figs. 4g and S31). Concurrently, the detection of N = N vibrational signatures confirms successful DAAT synthesis. Collectively, these findings establish a stepwise activation pathway: (1) Electric field-induced adsorption of hydroxyl groups forms reactive OH^*^ intermediates; (2) subsequent cleavage of N–H bonds generates -NH^*^ radicals; (3) radical coupling yields -NH-NH- intermediates that ultimately evolve into DAAT products while enabling anodic hydrogen production through this coupled proton–electron transfer process.

Building upon the demonstrated high-current capability of DATOR, we further integrated the DATOR||HER coupling system with an anion-exchange membrane water electrolyzer (AEMWE) to assess its industrial scalability. As schematically illustrated in Fig. 5g, the AEMWE prototype employed CuO/CF and Pt_s,n_@NiS_2_@CC as anode and cathode, respectively. LSV characterization revealed that the system requires merely 2.2 V to achieve an industrial-relevant current density of 1.0 A cm^−2^ (Fig. 5h). CP testing at 500 mA cm^−2^ confirmed exceptional operational stability, maintaining continuous hydrogen production for over 300 h with minimal degradation. Remarkably, the observed gradual potential drift during prolonged operation could be fully reversed by substrate replenishment, restoring the system to its initial performance level a critical feature for practical applications (Fig. 5i). Faradaic efficiency (FE) analysis demonstrated DATOR superior selectivity across varying current densities: FE remained above 90% at low current regimes (< 100 mA cm^−2^), while still maintaining 78.7% efficiency at 100 mA cm^−2^ (Fig. S41). These metrics collectively confirm the system’s industrial viability. The dual-electrode hydrogen production strategy inherently addresses the market imbalance between hydrogen demand and DAAT utilization, simultaneously enabling green synthesis of energetic materials through DAAT production and low-energy hydrogen generation a dual-output approach that establishes new paradigms for sustainable electrochemical manufacturing.

## Conclusions

This study successfully developed an electrochemical strategy based on a DATOR||HER coupled system, achieving synergistic production of hydrogen energy and high-nitrogen-content azo compounds under low-voltage driving. By constructing a bifunctional catalytic system comprising a Pt_s,n_@NiS_2_@CC cathode and CuO/CF anode, the system achieved a current density of 10 mA cm^−2^ at an ultra-low cell voltage of 0.96 V, demonstrating a 35.8% reduction in energy consumption compared to conventional water electrolysis. Theoretical calculations revealed that the synergistic effect between Pt single atoms and nanoparticles optimizes hydrogen adsorption energy (ΔG_H*_ = − 0.22 eV), while the OH^*^-mediated N–H activation pathway on the CuO anode significantly reduces the reaction energy barrier (0.17 eV), enabling efficient DAAT synthesis (FE > 90%) with simultaneous dual-electrode hydrogen evolution. Furthermore, the coupled system exhibited exceptional industrial-scale stability (> 300 h @500 mA cm^−2^) and reversibility in anion-exchange membrane electrolyzers, effectively addressing the product market capacity mismatch inherent in traditional organic oxidation coupling systems. This work not only provides a novel approach for green electrosynthesis of energetic materials but also establishes a theoretical foundation for integrated design of scalable hydrogen production and high-value chemical manufacturing. Future research should focus on extending this strategy to electrocatalytic synthesis of other organic molecules and renewable energy storage applications.

## Supplementary Information

Below is the link to the electronic supplementary material.

Supplementary file1 (DOCX 7851 kb)
