# Supplementary material for: Electrocatalytic Self-Coupling of N-Heterocyclic Amides for Energy-Efficient Bipolar Hydrogen Production
Source: Nanomicro Lett. 2026 Jan 4;18:197. doi: 10.1007/s40820-025-02025-3 (PMC12765757; doi:10.1007/s40820-025-02025-3)
Supplement: Supplementary file 1 — Supplementary file1 (DOCX 7851 kb) [file 40820_2025_2025_MOESM1_ESM.docx]

Supporting Information for

**Electrocatalytic Self-Coupling of N-Heterocyclic Amides for Energy-Efficient Bipolar Hydrogen Production**

Yuqiang Ma^1^, Meng Li^1^, Dandan Zhang^1^, Cihang Wang^1^, Yu Li^1^, Zihang Zhao^1^, Xiaogang Mu^2^, Jun Hu^1^ , Xiang Hu^3^*, Jiachen Li^1^*, Haixia Ma^1, 2^*, Zhenhai Wen^3^

^1^ Xi'an Key Laboratory of Special Energetic Materials, School of Chemical Engineering, Northwest University, Xi’an 710127, P. R. China

^2^ Zhijian Laboratory, Xi’an 710025, P. R. China

^3^ State Key Laboratory of Structural Chemistry, and Fujian Provincial Key Laboratory of Materials and Techniques toward Hydrogen Energy, Fujian Institute of Research on the Structure of Matter, Chinese Academy of Sciences, Fuzhou, Fujian 350002, P. R. China

*Corresponding authors. E-mail: [mahx@nwu.edu.cn](mailto:mahx@nwu.edu.cn) (Haixia Ma); [huxiang@fjirsm.ac.cn](mailto:huxiang@fjirsm.ac.cn) (Xiang Hu); [lijiachen@nwu.edu.cn](mailto:lijiachen@nwu.edu.cn) (Jiachen Li)

**S1 Electrochemically active surface area (ECSA) calculation**

The ECSA of the catalysts layer can be calculated as:

$$ECSA=\frac{C_{dl}}{C_{s}}$$

Where the specific capacitances (C_s_) were chosen as C_s_ = 0.040 mF cm^−2^ in 1.0 M KOH. C_dl_ was derived as the average value of linear fitted slope by plotting both the anodic and cathodic current against the scan rate from the corresponding cyclic voltammetry (CV) curves at non-faradic potential.

**S2 Pt mass activity calculation method**

Because Pt species were supported by NiS_2_@CC in the measurements, the contributions of the NiS_2_@CC should be deducted. Therefore, the Pt mass activities of Pt_s,n_@NiS_2_@CC can be calculated based on Fig. 2g using the following equation:

Pt mass activity of Pt_s,n_@NiS_2_@CC:

$$J_{mass}^{Pt}=\frac{J_{area}^{{Pt}_{s,n}@{NiS}_{2}@CC}-J_{area}^{{NiS}_{2}@CC}}{{mass}_{Pt}}$$

**S3 TOF calculated method**

The conversion efficiency of Pt_s,n_@NiS_2_@CC was evaluated by the TOF values (s^–1^), which can be obtained according to the Eq:

TOF=$\frac{Total numberofH_{2} turnover/geometric area ({cm}^{2})}{Total number of activesites/geometric area({cm}^{2})}$

The total number of H_2_ turnovers were calculated by the Eq:

Total H_2_ turnovers =

[J (mA cm^-2^)]$\left[ \frac{1C}{{10}^{3}\left( mA \right)} \right]\left[ \frac{1\left( mol e^{-} \right)}{96485\left( C \right)} \right]\left[ \frac{1 mol H_{2}}{2 mol e^{-}} \right][\frac{6.02\times{10}^{23}molecules H_{2}}{1 mol H_{2}}]$

The number of active sites was calculated by the total mass of the Pt quantified by the ICP-OES. The current density (J) from LSV can be converted to TOF according to the Eq:

TOF=$\frac{3.12\times{10}^{15}}{Number of active sites}\times$|J| (s^-1^)

**S4 Electricity input (W) of the coupling system per m^3^ of H_2_ produced (kWh per m^3^ H_2_) was calculated method**

$$W=\frac{n\times F\times U\times1000}{3600\times V_{m}}$$

Where the n is the number of electrons transferred for H_2_ production (n = 2), U is the applied cell voltage in the two-electrode cell, *V_m_* is the molar volume of H_2_ at normal temperature and pressure (22.4 mol L^−1^ ), F is Faraday constant (96485 C mol^−1^ ).

**S5 Experimental Section**

***Preparation of Ni(OH)_2_@CC:*** The carbon cloth (CC, 1 × 3 cm) substrate underwent sequential purification via ultrasonic cleaning in acetone, ultrapure water, and ethanol (10 min each) to eliminate surface oxides and insoluble contaminants. Subsequently, a precursor solution was formulated by dissolving Ni(NO_3_)_2_·6H_2_O (0.20 g), urea (CH_4_N_2_O, 0.23 g), and NH_4_F (70 mg) in 15 mL deionized water under vigorous stirring. This homogeneous mixture was transferred into a Teflon-lined autoclave containing the pretreated CC, followed by hydrothermal treatment at 120 °C for 6 h to in situ grow Ni(OH)_2_ nanostructures on the carbon framework. The resulting Ni(OH)_2_/CC composite was collected, rinsed thoroughly, and dried at 60 °C to serve as the precursor for subsequent sulfidation processes.

***Material characterizations:*** Scanning electron microscopy (SEM) images of the samples were obtained by Hitachi, Su8010. Transmission electron microscopy (TEM), high-resolution TEM (HRTEM) and element mapping was performed using FEI Talos F200X microscopy. XPS was performed on the Thermo Scientific Nexsa spectrum. The microstructure of the samples was analyzed using an FEI Themis Z transmission electron microscope equipped with spherical aberration correction (AC-TEM). The X-ray powder diffraction (XRD) patterns were recorded using a Bruker D8 Advance. X-ray absorption spectroscopy (XAS) measurements were performed at beamline 1W1B of the Beijing Synchrotron Radiation Facility (BSRF), where the storage ring operates at a typical energy of 2.5 GeV. The spectra were acquired in fluorescence mode with energy calibration established using standard Pt foil and PtO_2_ reference materials. Chemical composition analysis was performed using an Agilent 5110 inductively coupled plasma optical emission spectrometer (ICP-OES). The ¹H and ¹³C nuclear magnetic resonance (NMR) spectra were recorded on a Bruker 400 MHz NMR spectrometer. X-ray photoelectron spectroscopy (XPS) was performed with Thermo Scientific Nexsa. Raman spectra were obtained by Thermo DXR green laser (532 nm). The deuterated DAT substrates and DATOR products were characterized by ¹³C and ¹H nuclear magnetic resonance (NMR) spectroscopy using a Bruker Advance 400 MHz spectrometer, with measurements conducted in deuterium oxide (D_2_O) at 25 °C.

***In situ attenuated total reflection Fourier-transform infrared (ATR-FTIR):*** The reaction mechanism of DATOR was probed using in situ Fourier transform infrared spectroscopy (FTIR) conducted on a Nicolet IS50 FTIR spectrometer equipped with a mercury cadmium telluride (MCT) cryogenic detector, coupled with a Pike Technologies VeeMAX III attenuated total reflectance (ATR) accessory and Jackfish J1 electrochemical cell. The working electrode was fabricated by depositing a 50 nm gold film via vacuum sputter deposition onto a silicon ATR crystal, serving as an architectural support for CuO/CF catalyst immobilization. The CuO NW catalyst ink was prepared through ultrasonic exfoliation of CuO/CF (1 cm^2^ geometric area) in a ternary solvent system comprising: Ethanol (0.5 mL, ≥ 99.7%), Deionized water (0.5 mL, 18.2 MΩ·cm) and Nafion ionomer solution (40 μL, 5 wt% in lower aliphatic alcohols, Dupont D520).The homogeneous colloidal suspension was subsequently deposited onto the gold-coated substrate via micro-syringe drop-casting followed by ambient drying at 25 ℃ under relative humidity < 40%.

***In situ differential electrochemical mass spectrometry (DEMS):*** The gaseous products generated during the anodic DATOR process were analyzed using an in situ differential electrochemical mass spectrometry (DEMS) system (Shanghai Linglu Instrument Equipment) under ultrahigh vacuum conditions (< 10^−7^ Pa). The electrochemical cell and vacuum chamber were physically isolated by a microporous polytetrafluoroethylene (PTFE) membrane, enabling selective gas permeation while effectively preventing electrolyte contamination. Trace-level gaseous species were quantitatively monitored through real-time mass spectrometric detection integrated with the electrochemical setup.

***Electrochemical characterizations:*** All electrochemical measurements were conducted using a CHI760e electrochemical workstation (Chenhua, Shanghai). The hydrogen evolution reaction (HER) performance was evaluated in a three-electrode configuration, where the as-prepared Pt_s,n_@NiS_2_@CC served as the working electrode, with high-purity graphite and Hg/HgO electrodes functioning as the counter electrode and reference electrode, respectively. The electrolyte consisted of 1.0 M KOH aqueous solution. Linear sweep voltammetry (LSV) was performed within a potential window of -0.6 to -1.5 V (vs. Hg/HgO) at a scan rate of 2 mV s^−1^. The catalytic stability was further assessed through cyclic voltammetry (CV) and chronopotentiometric (CP) measurements. For the anodic DATOR system characterization, a three-electrode setup was employed using CuO/CF as the working electrode, Pt wire as the counter electrode, and Hg/HgO as the reference electrode. The electrolyte solutions contained 1.0 M KOH with varying DAT concentrations (0.1, 0.2, 0.3, 0.4 M). LSV tests were conducted in the potential range of 0.6-1.8 V (vs. RHE). All measured potentials in three-electrode systems were subjected to IR compensation using the following equation:

$$E (vs. RHE) = E (vs. Hg/HgO)+ 0.059 \times pH + 0.098$$

The HER||DATOR coupling system was tested in a two-electrode system completed in an H-type electrolyzer. Pt_s,n_@NiS_2_@CC and CuO/CF were used as the cathode and anode, respectively, with a working area of 0.25 cm^−2^. The cathode and anode were divided into two chambers in the middle by an anion exchange membrane (FumasepFAA-3-50). The cathode and anode chambers used 1.0 M KOH and 1.0 M KOH + 0.2 M DAT as electrolytes, respectively. The AEMWE device also used the same electrode types as described above, except that the working area of both electrodes was 1.0 cm^−2^. The AEMWE device was operated with a CHI760e (Brilliance) equipped with a current amplifier, using 1.0 M KOH and 1.0 M KOH + 0.2 M DAT as the cathodic and anodic electrolytes, respectively, at room temperature. MKOH and 1.0 M KOH + 0.2 M DAT, respectively, for cathode electrolyte and anode electrolyte to study the flow cell performance. The separation of DAAT products was based on the difference in solubility. The pH was adjusted to neutral with sulfuric acid, stirred at 100 °C for 30 min, and treated with a thermal filter. The stability tests were conducted via chronopotentiometry at a current density of 500 mA cm^−2^ for 500 h. Due to the rapid consumption of the DAT substrate and water during the prolonged stability evaluation, the electrolyte was subjected to irregular replenishment to maintain consistent reaction conditions.

***Theoretical calculations:*** Density functional theory (DFT) calculations were performed using the Vienna Ab initio Simulation Package (VASP 6.4.2) with the projector augmented wave (PAW) pseudopotential method. The electron exchange-correlation interactions were treated within the generalized gradient approximation (GGA) using the Perdew-Burke-Ernzerhof (PBE) functional. A plane-wave basis set with an energy cutoff of 500 eV was employed to expand the Kohn-Sham wavefunctions, ensuring convergence of the electronic structure calculations. The Brillouin zone integration was sampled using a Γ-centered 3 × 3 × 1 k-point mesh generated through the Monkhorst-Pack scheme. Electronic self-consistency was achieved through iterative solution of the Kohn-Sham equations using the self-consistent field (SCF) method, with convergence criteria set to 1 × 10^−5^ eV for total energy differences. Structural optimizations were performed using the conjugate gradient algorithm, requiring atomic forces to converge below 0.05 eV/Å and lattice stresses within 0.1 GPa. Van der Waals interactions between adsorbates and catalyst surfaces were explicitly considered through Grimme DFT-D3 dispersion correction method. Reaction pathways were investigated using the climbing image nudged elastic band (CI-NEB) method to identify transition states and minimum energy paths. Thermodynamic properties were calculated through vibrational frequency analysis, with zero-point energy (ZPE) corrections and entropy contributions evaluated in the harmonic approximation. The Gibbs free energy change (ΔG) was determined using the relation:

$$\Delta G=\Delta E_{ads}+\Delta E_{ZPE}-T\Delta S$$

where ΔEads is the difference between the energies of the product and reactant molecules adsorbed on the surface of the catalyst, calculated by DFT; ΔEZPE and ΔS are the zero-point energy and entropy changes obtained from the vibrational frequency, respectively.

The adsorption energy (ΔEads) is defined as:

$$\Delta E_{ads}=E_{(total)}-E_{(surface)}-E_{(adsorbate)}$$

E(total) is the total energy of the adsorbate and the catalyst, then E(surface) is the energy of the catalyst and E(adsorbate) is the energy of the adsorbate.

**Supplementary Figures and Tables**


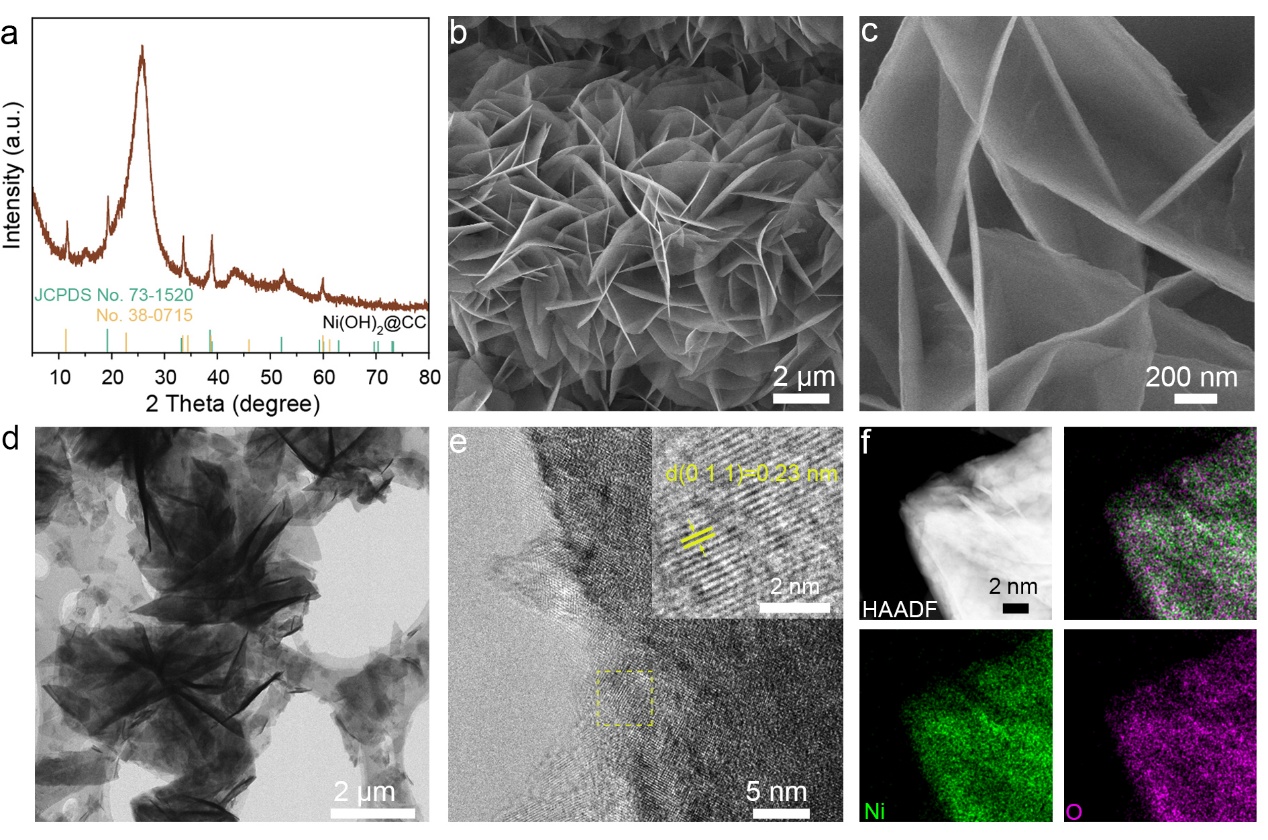


**Fig. S1** **a**) XRD patterns of Ni(OH)_2_. **b, c**) SEM and **d, e**) TEM images of Ni(OH)_2_. HAADF-STEM image and elemental mappings of O and Ni, respectively, for Ni(OH)_2_


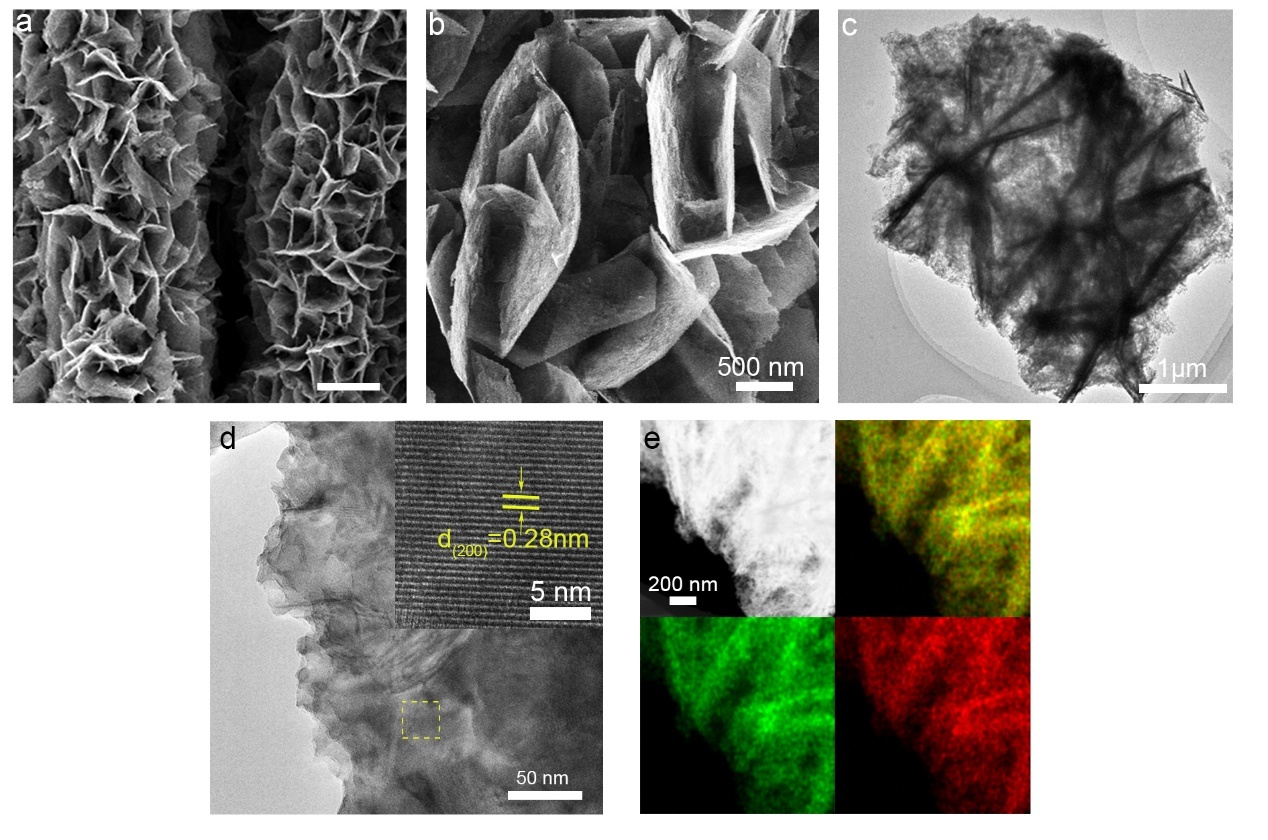


**Fig. S2** a, b) SEM and c, d) TEM images of NiS_2_. HAADF-STEM image and elemental mappings of S and Ni, respectively, for NiS_2_


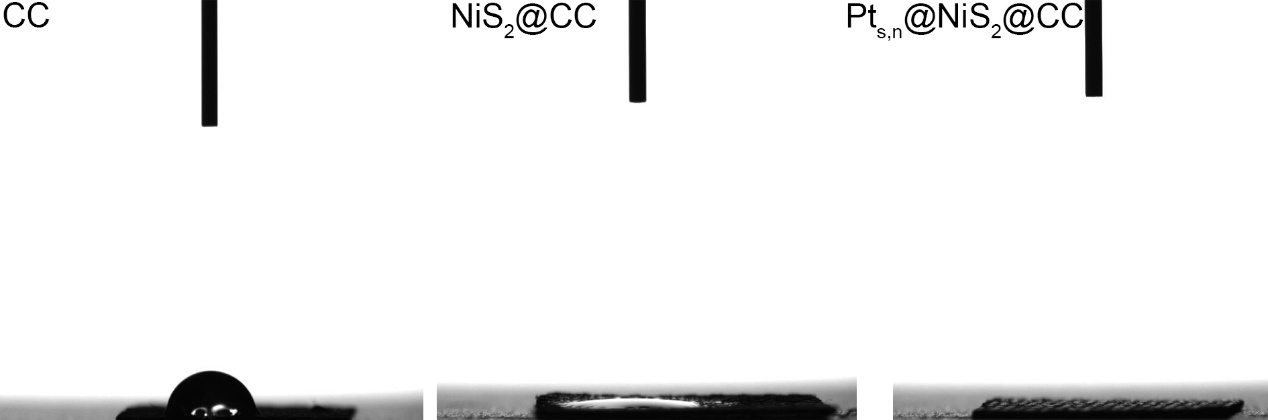


**Fig. S3** Contact angle of water droplets sitting on the surface of the a) CC substrate, b) NiS_2_@CC, c) Pt_s,n_@NiS_2_@CC


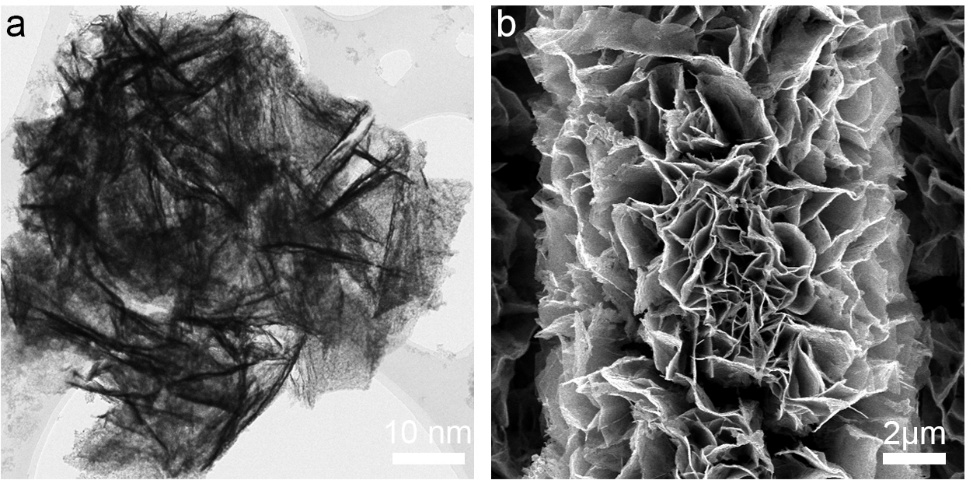


**Fig. S4** Low resolution **a**) TEM and **b**) SEM of Pt_s,n_@NiS_2_@CC


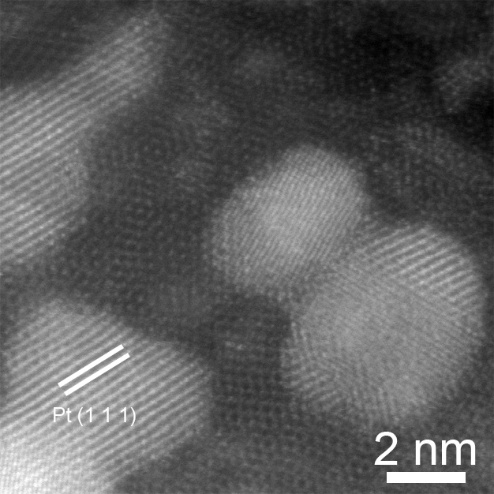


**Fig. S5** AC-HAADF STEM images of Pt_s,n_@NiS_2_@CC


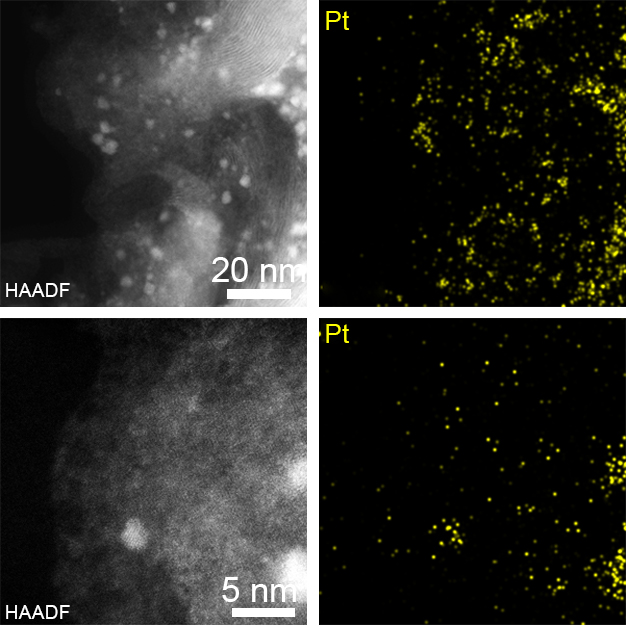


**Fig. S6** AC-STEM-HAADF images and Pt mapping of Pt_s,n_@NiS_2_@CC


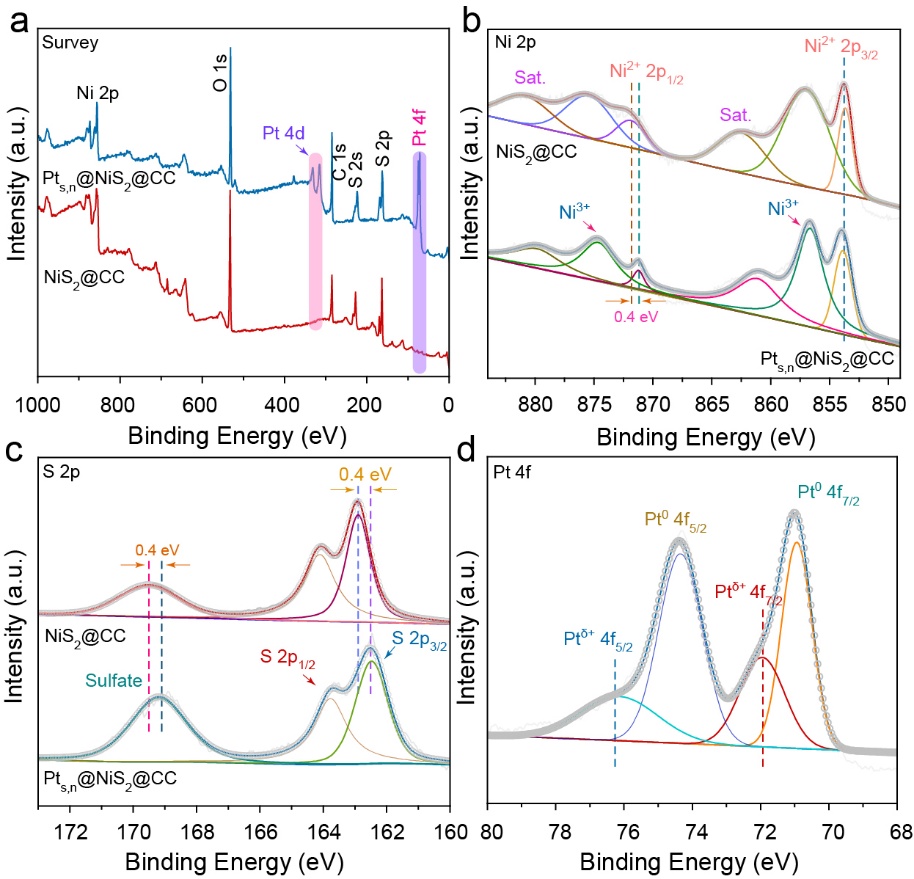


**Fig. S7** **a**) XPS survey spectra of NiS_2_@CC and Pt_s,n_@NiS_2_@CC. **b**) Ni 2p and **c**) S 2p XPS spectra of NiS_2_@CC and Pt_s,n_@NiS_2_@CC. **d**) Pt 4f XPS spectra of Pt_s,n_@NiS_2_@CC


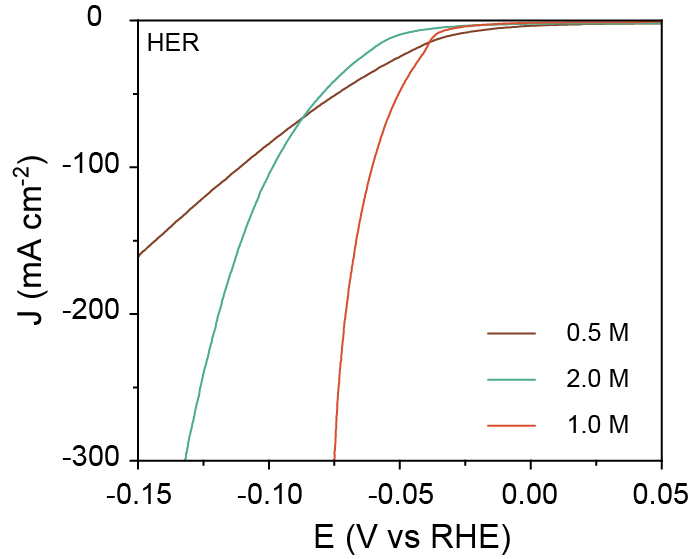


**Fig. S8** HER performance curves at different KOH concentrations (0.5 M and 2.0 M)


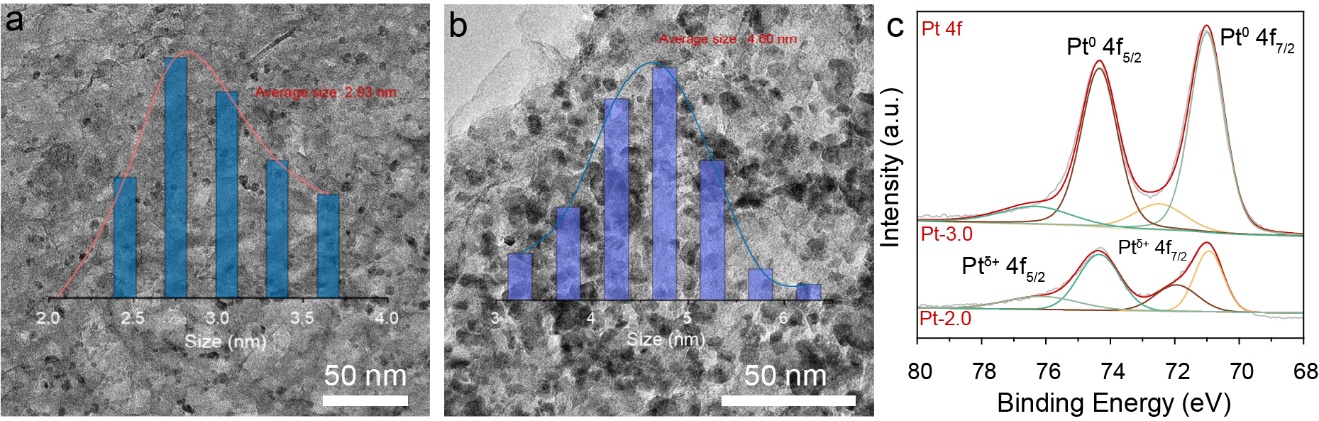


**Fig. S9** TEM images and particle size distribution of **a**) Pt-2.0 and **b**) Pt-3.0. **c**) Pt 4f XPS spectra of Pt-2.0 and Pt-3.0


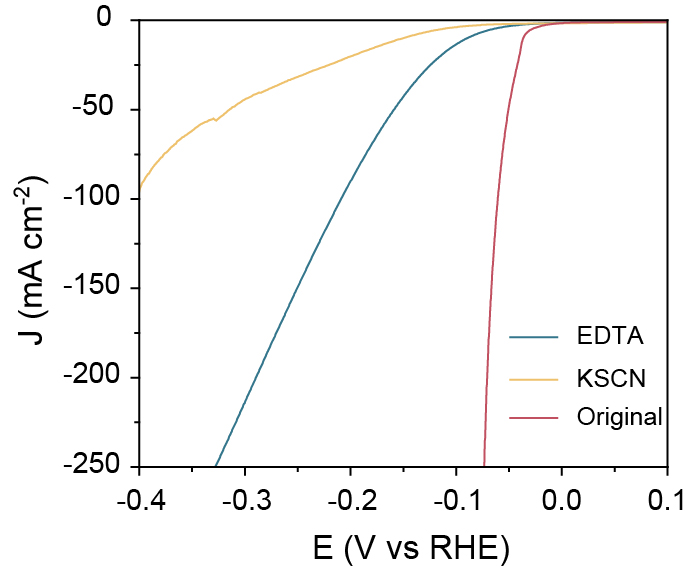


**Fig. S10** HER with and without 10 mM KSCN and 10 mM EDTA in the alkaline electrolytes


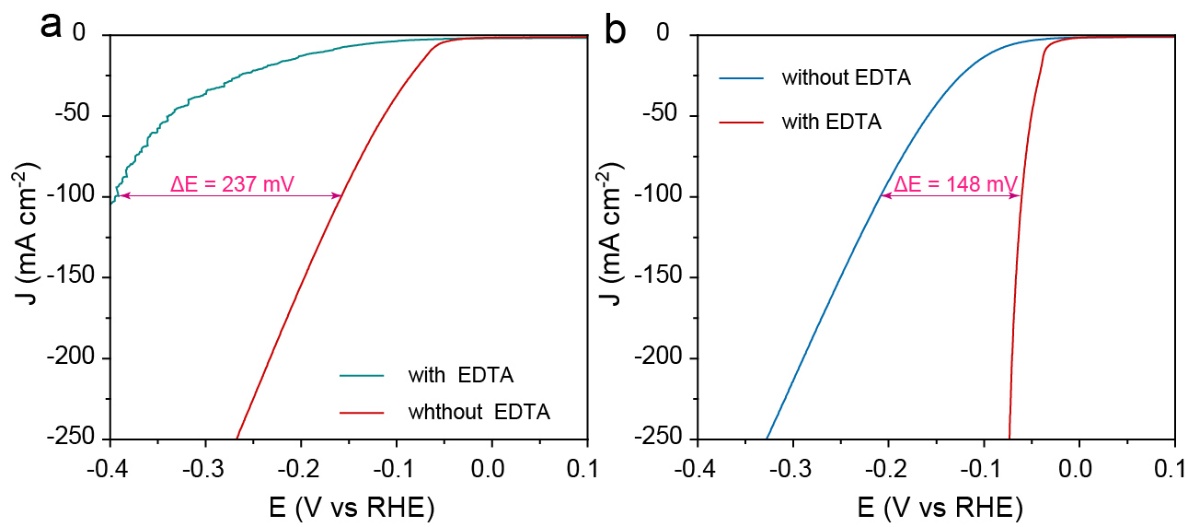


**Fig. S11** **a**) Pt-0.1 and **b**) Pt-2.0 of HER performance with and without 10 mM EDTA in the alkaline electrolytes


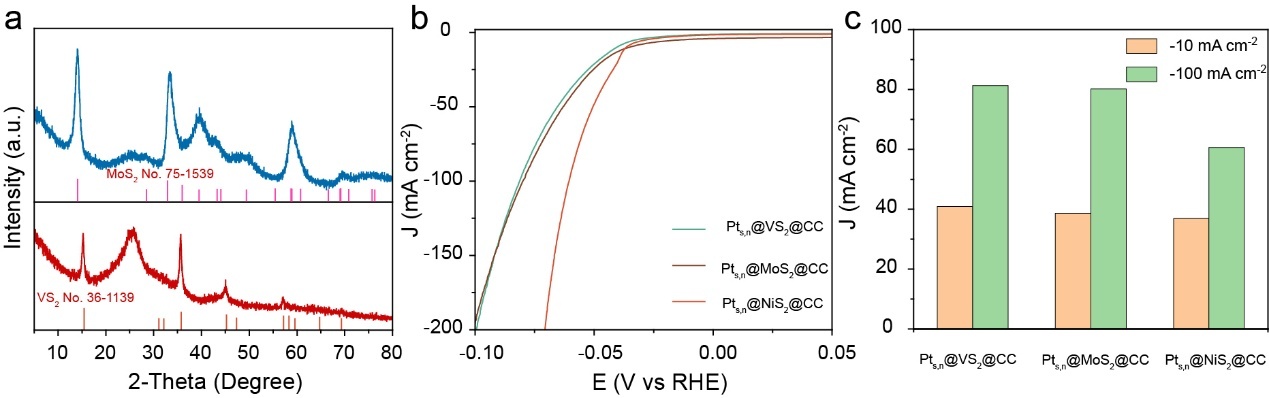


**Fig. S12** a) XRD patterns of MoS_2_@CC and VS_2_@CC. b) HER performance of difference samples. c) Comparison of the overpotential at −100 mA cm^−2^ and −10 mA cm^−2^ for different samples


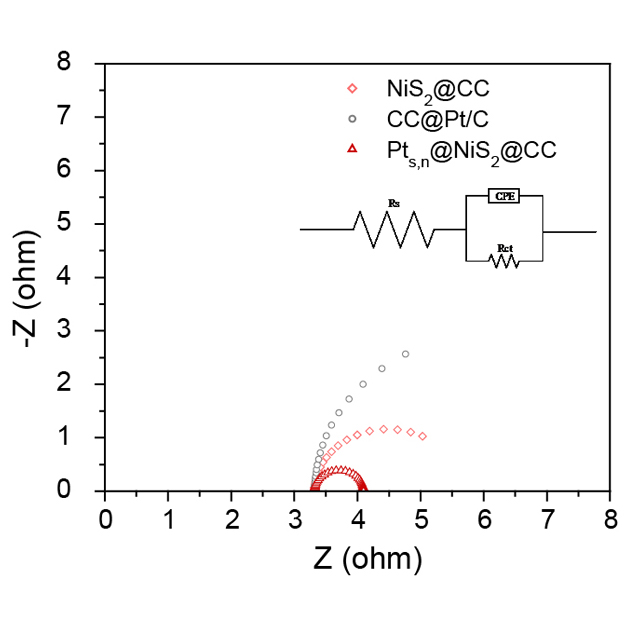


**Fig. S13** EIS Nyquist plots of NiS_2_@CC, Pt_s,n_@NiS_2_@CC and CC@Pt/C

**
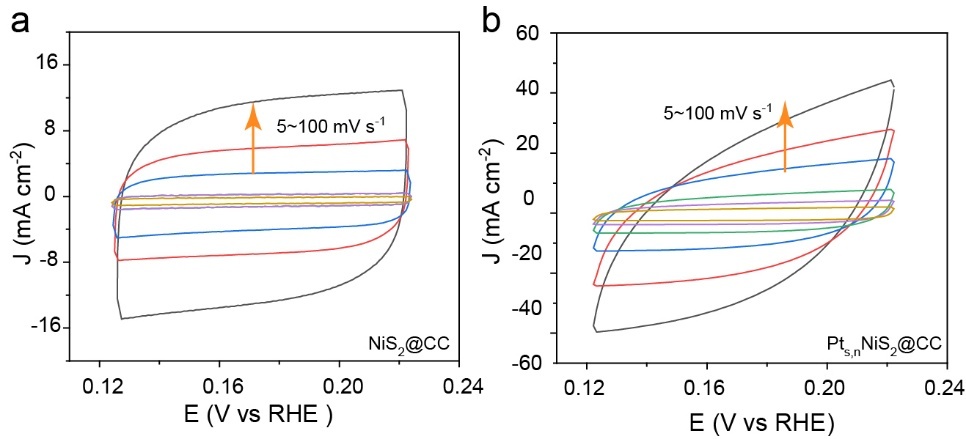
**

**Fig. S14** CV curves of a)NiS_2_@CC and b) Pt_s,n_@NiS_2_@CC over anon-faradic potential range with various scan rates of 2.5, 5, 10, 25, 50 and 100 mV s^-1^


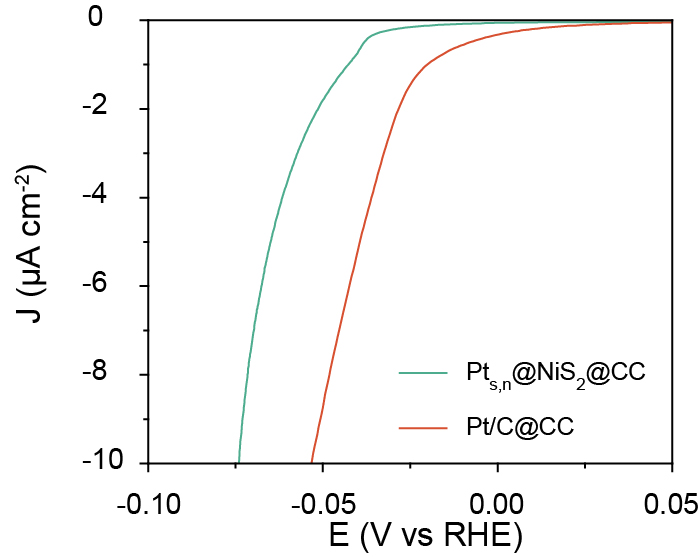


**Fig. S15** Normalized Fitted HER Performance Curve


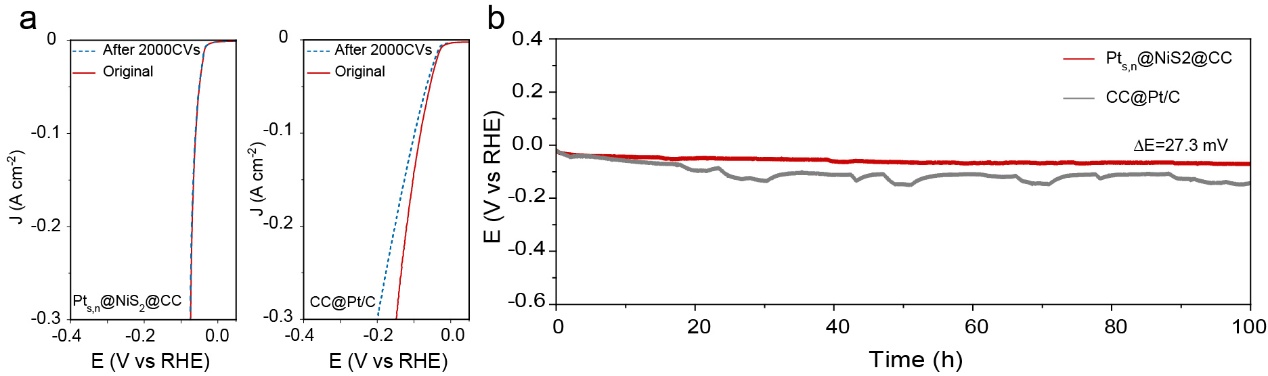


**Fig. S16** **a**) LSV curve of initial and after 2000 CVs cycle. **b**) CP test of Pt/C and Pt_s,n_@NiS_2_@CC


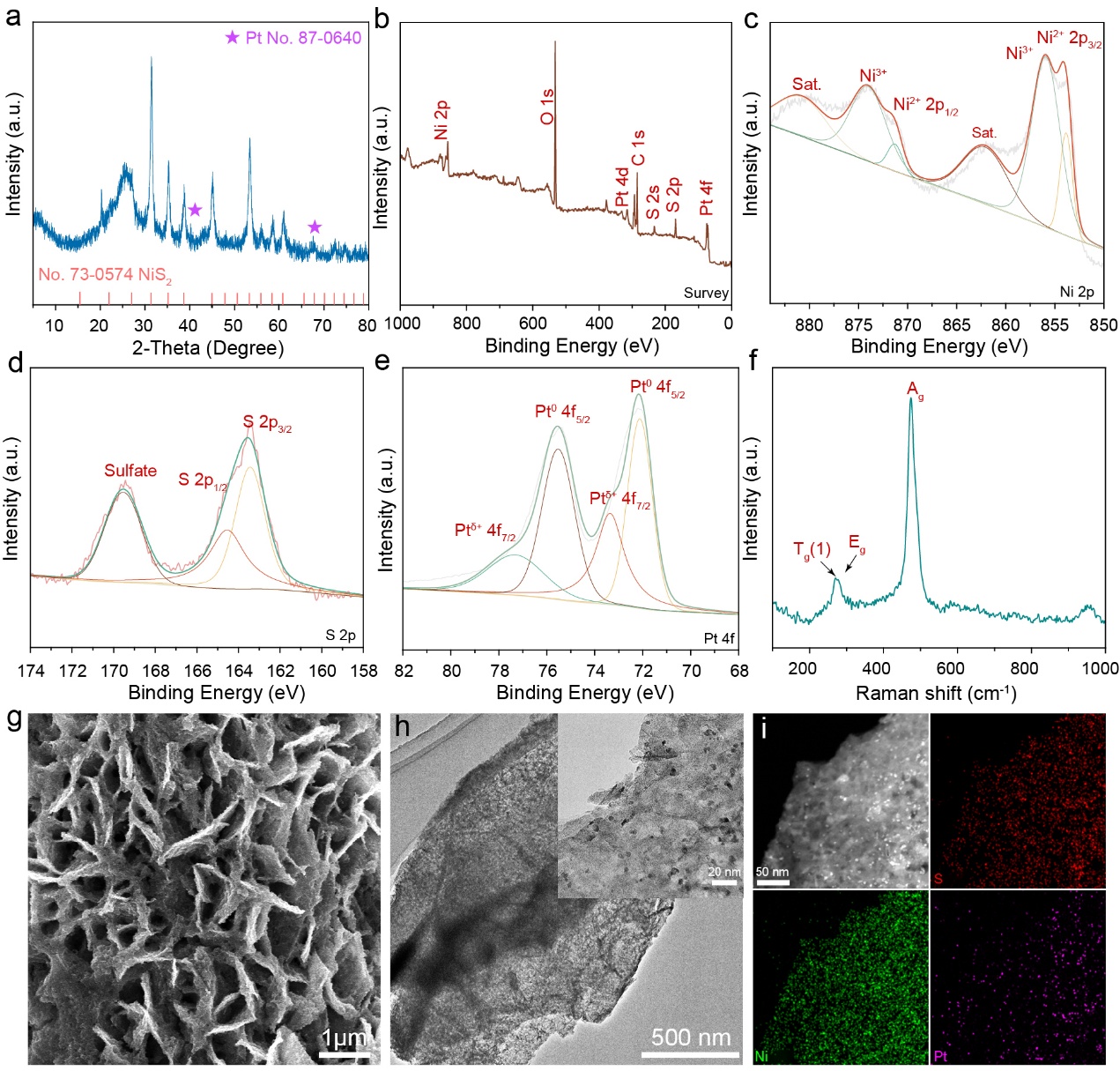


**Fig. S17** Structural and morphological characterizations of Pt_s,n_@NiS_2_@CC after the CP test. a) XRD patterns of Pt_s,n_@NiS_2_@CC. b) XPS survey spectrum and High-resolution spectra of (c) Ni 2p, (d) S 2p and (e) Pt 4f signals for Pt_s,n_@NiS_2_@CC after CP test. f) Raman spectra of Pts,n@NiS_2_@CC after CP test. g) SEM images of Pt_s,n_@NiS_2_@CC. h) Low and high-resolution TEM images of Pt_s,n_@NiS_2_@CC. i) Elemental mappings of Ni, Pt and S respectively, for Pt_s,n_@NiS_2_@CC


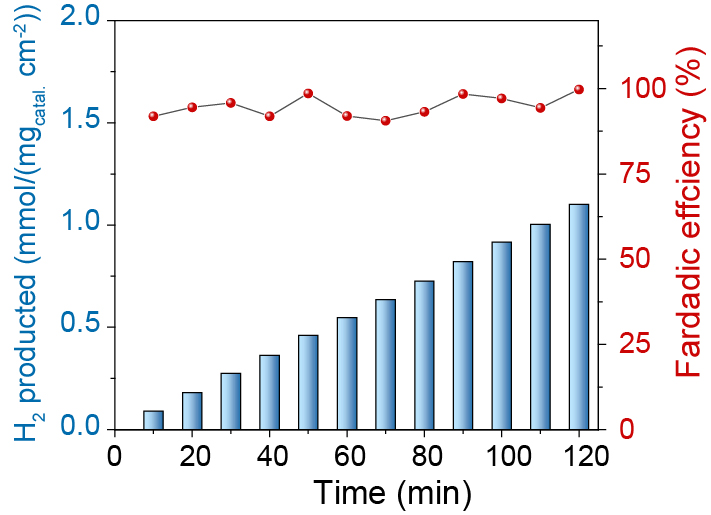


**Fig. S18** HER hydrogen production efficiency and Faraday electron efficiency


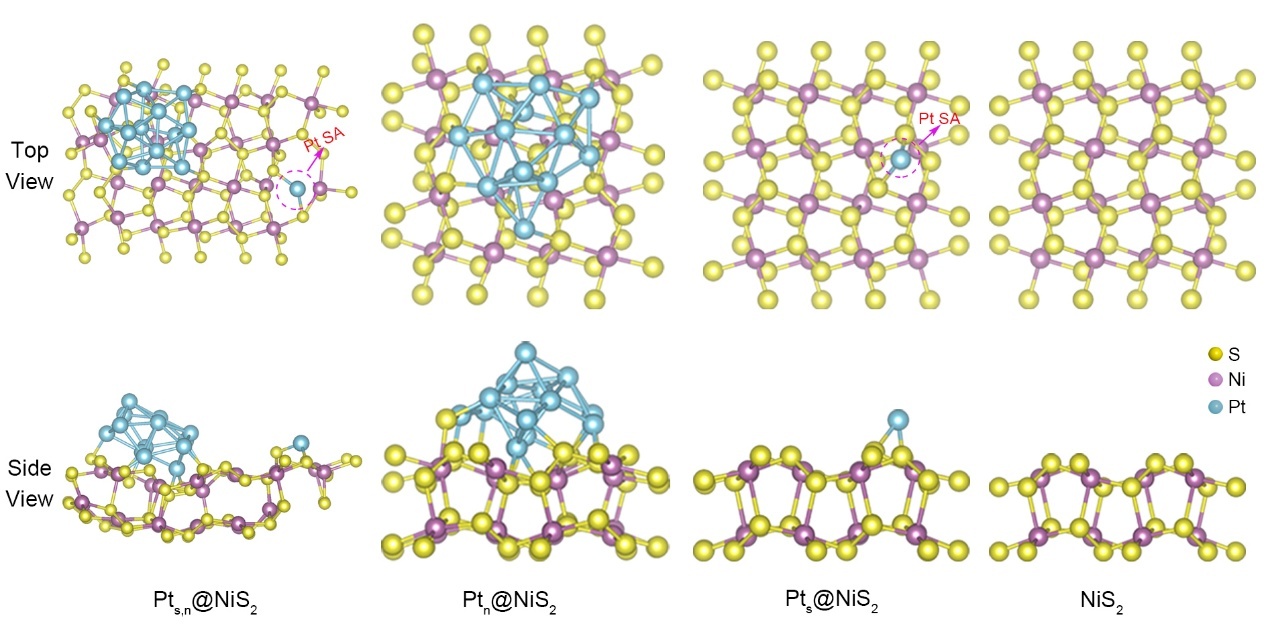


**Fig. S19** Theoretical model diagram for Pt_s_, Pt_n_ and Pt_s,n_ load NiS_2_


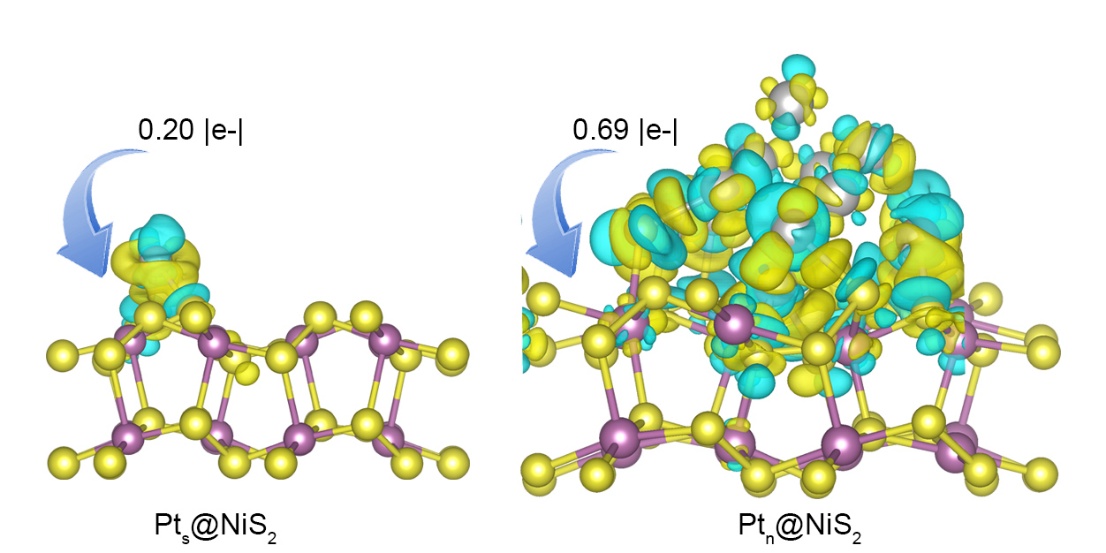


**Fig. S20** Differential charge density diagram of Pt_s_@NiS_2_ and Pt_n_@NiS_2_


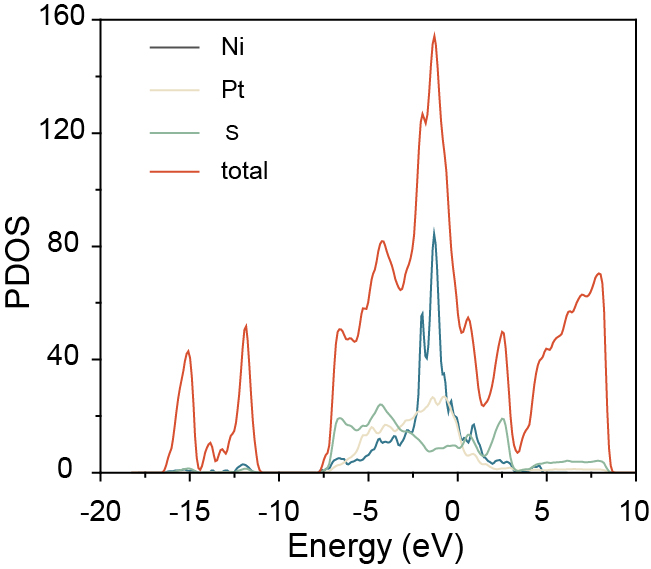


**Fig. S21** Pt_s,n_@NiS_2_@CC total element DOS diagram


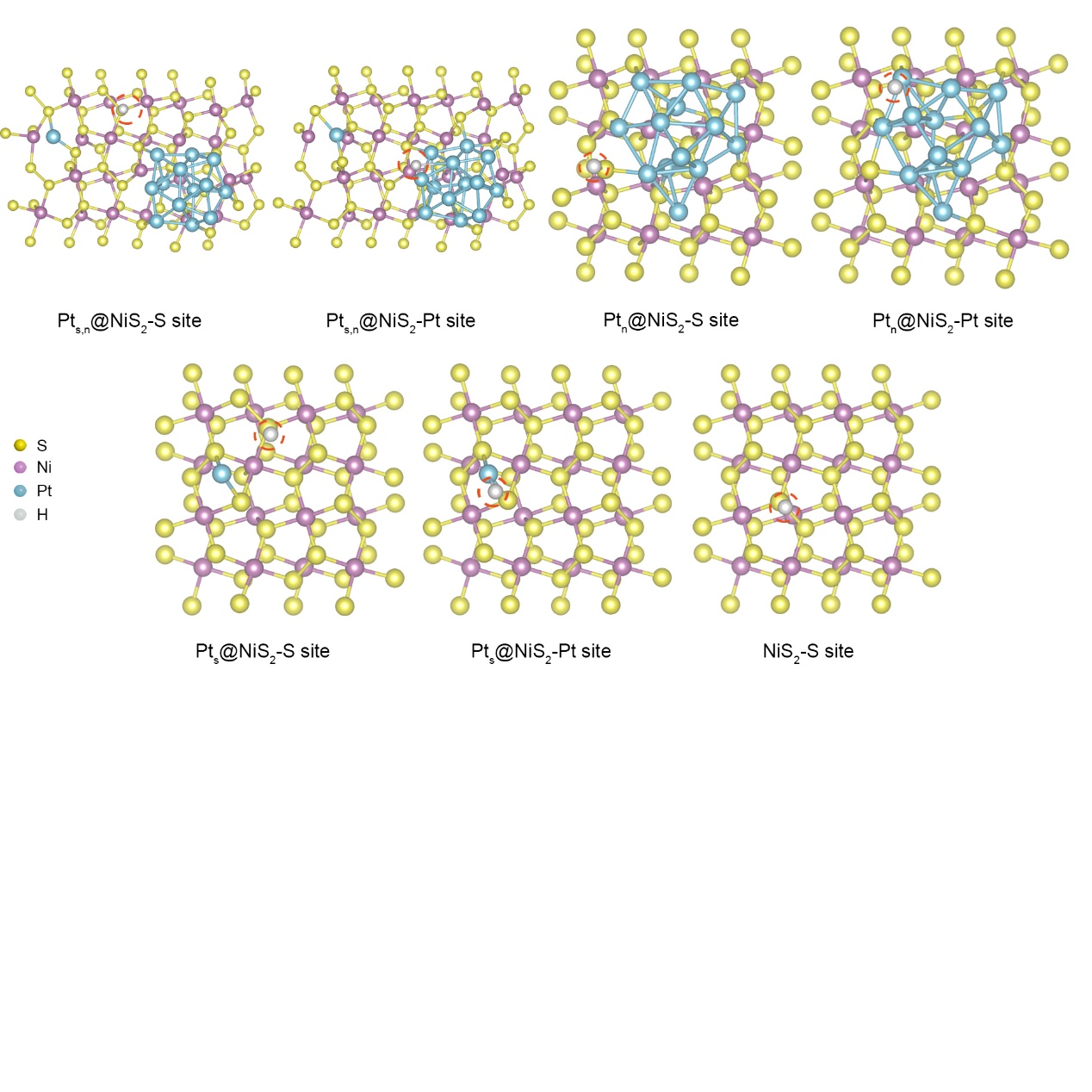


**Fig. S22** H* (red cycle) adsorption model diagram


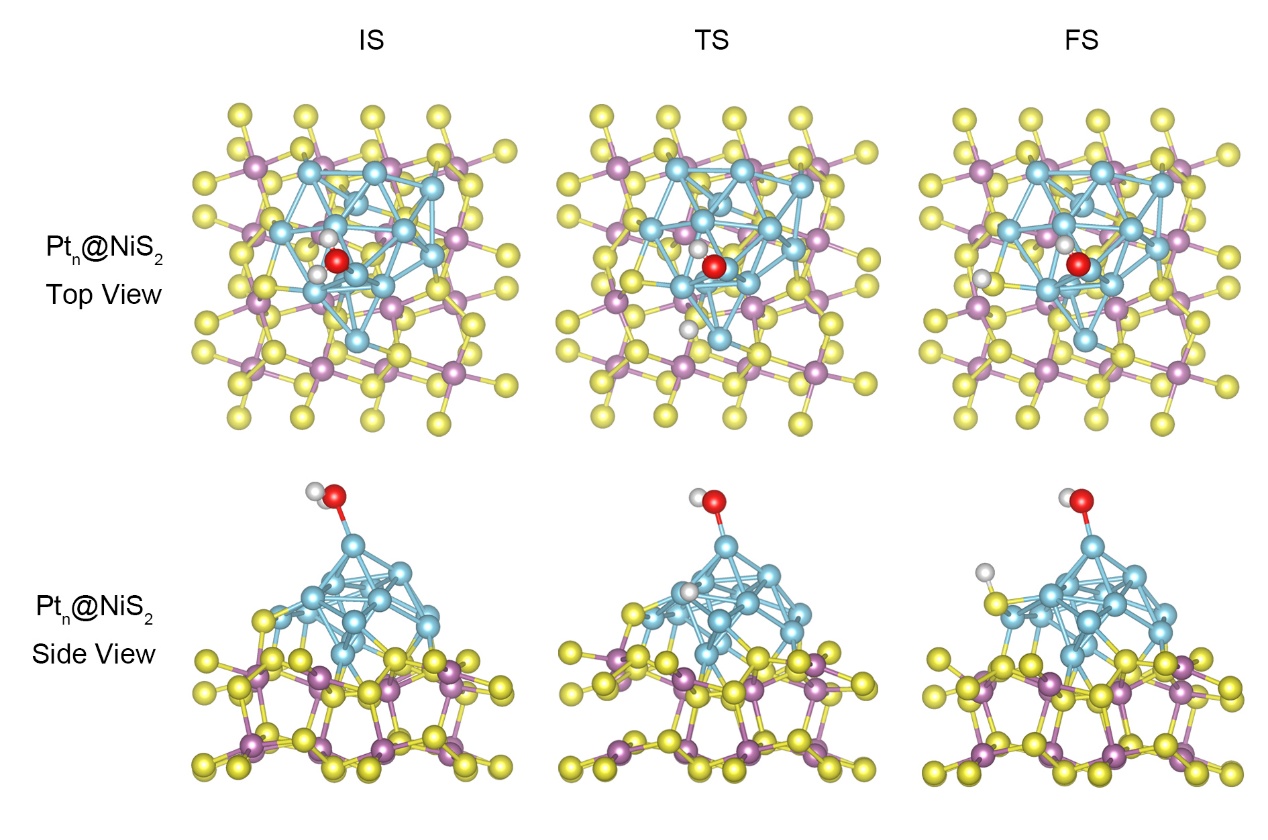


**Fig. S23** Structural models of initial state, transition state, and final state of Volmer pathway on Pt_n_@NiS_2_@CC


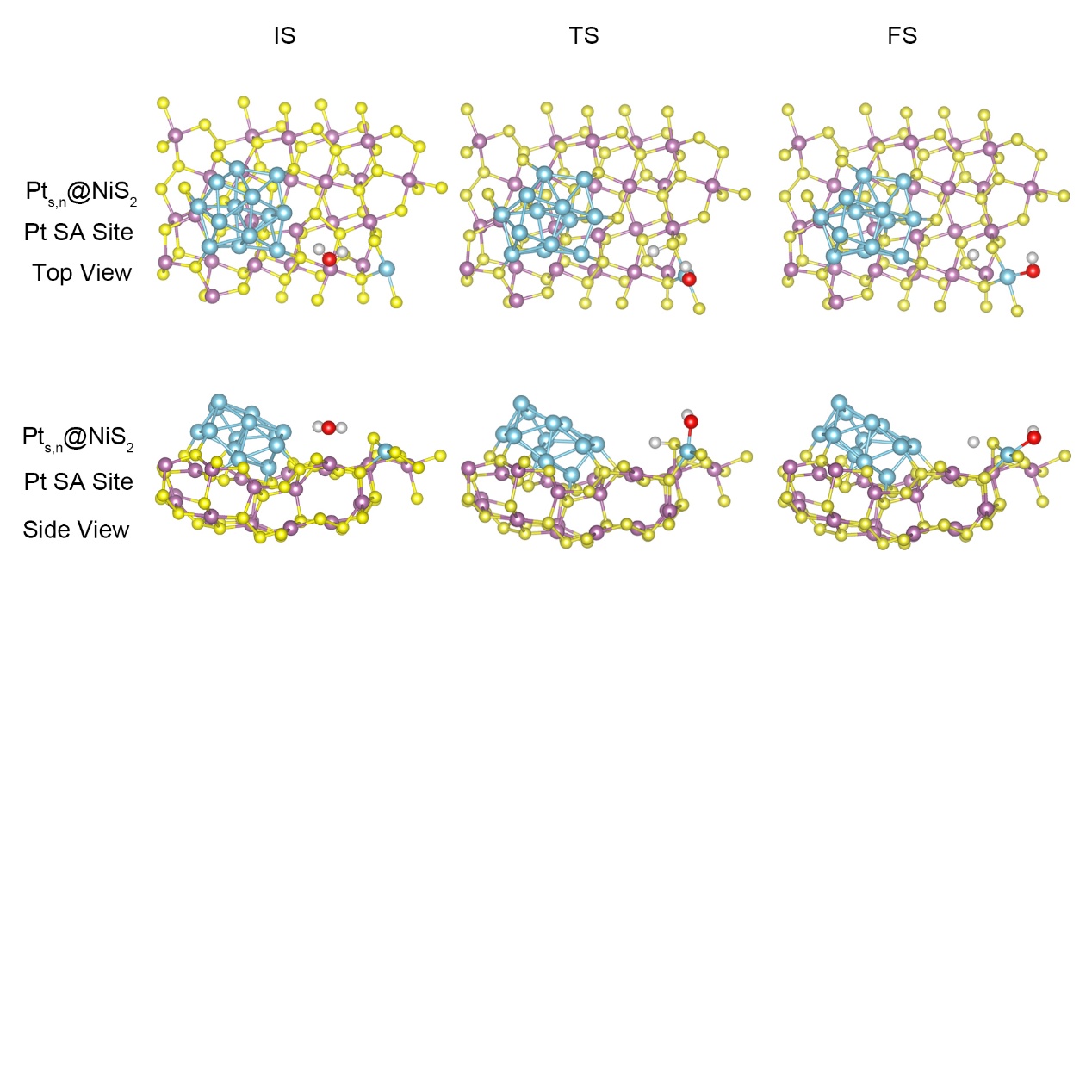


**Fig. S24** Structural models of initial state, transition state, and final state of Volmer pathway on Pt_s,n_@NiS_2_@CC Pt SA Site


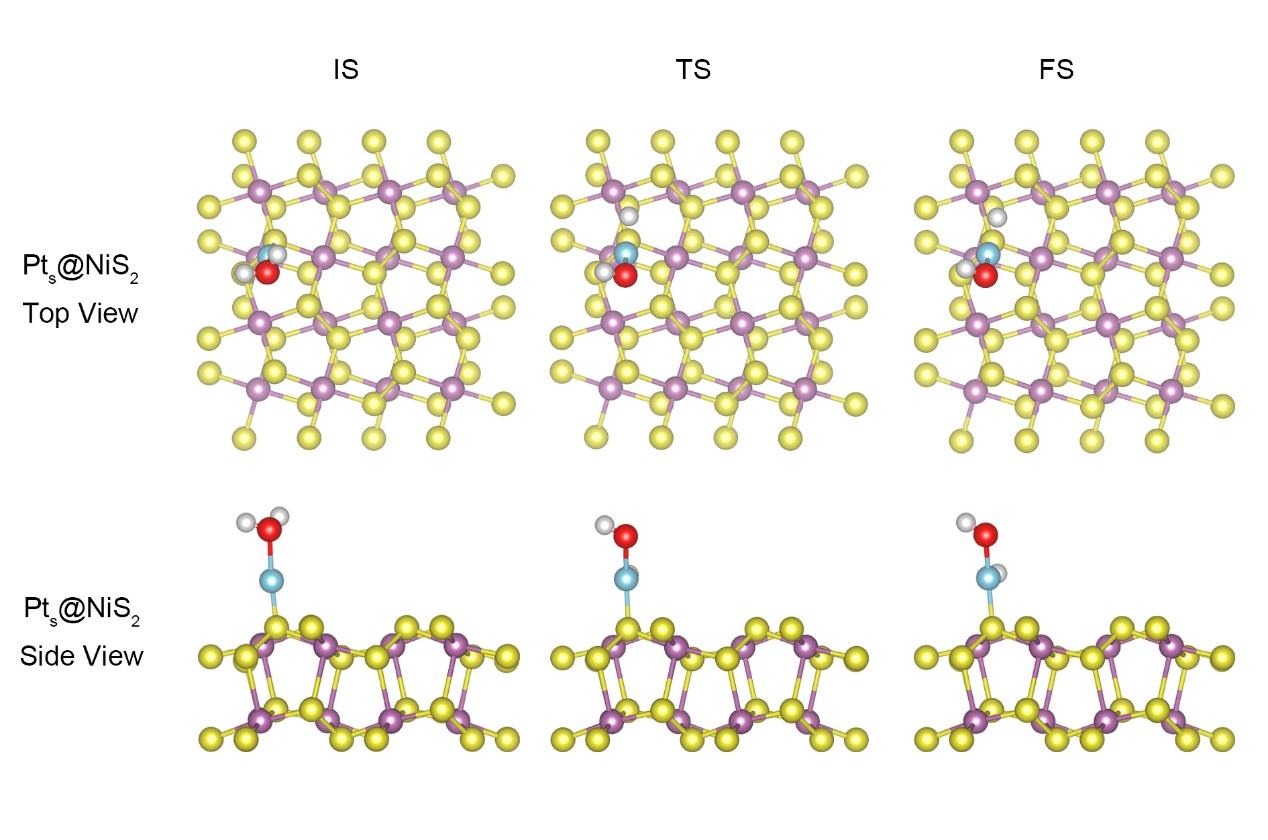


**Fig. S25** Structural models of initial state, transition state, and final state of Volmer pathway on Pt_s_@NiS_2_@CC


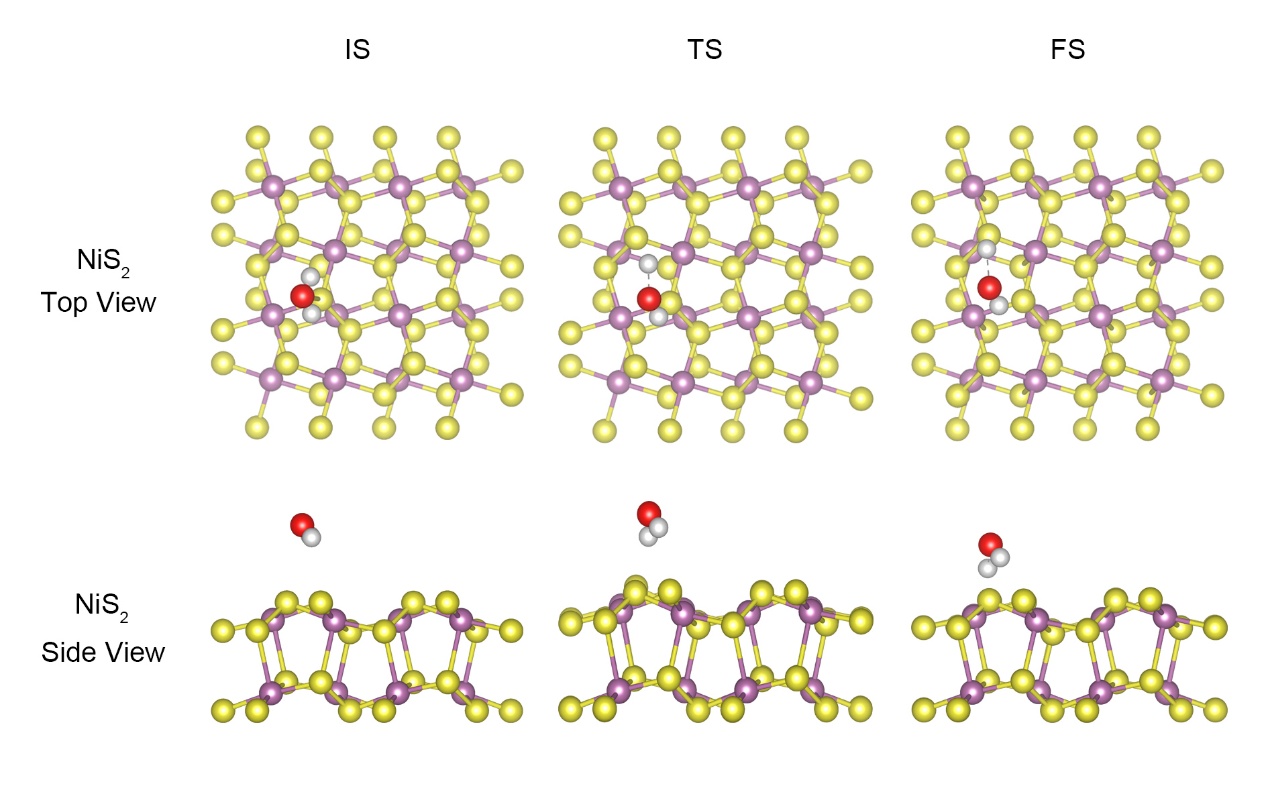


**Fig. S26** Structural models of initial state, transition state, and final state of Volmer pathway on NiS_2_@CC


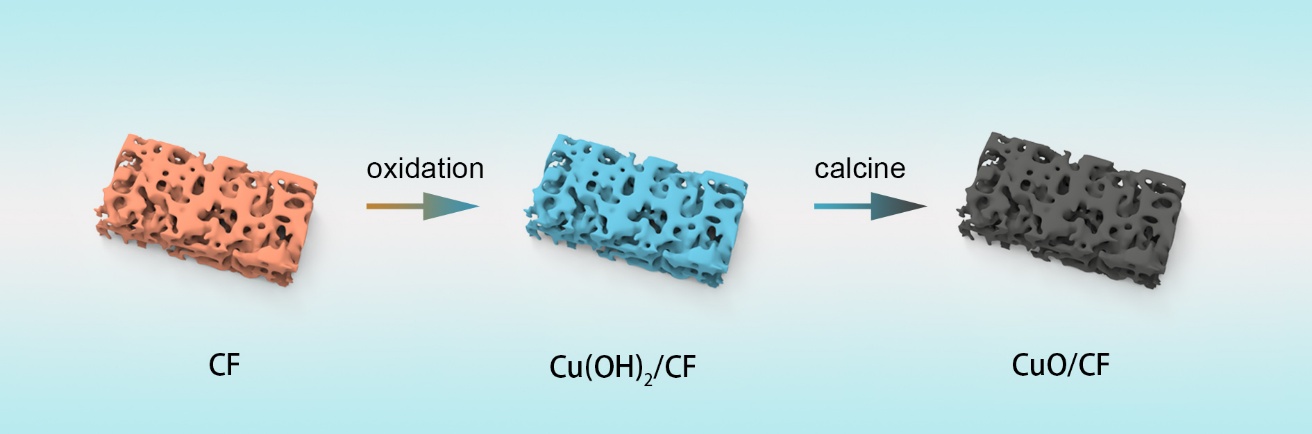


**Fig. S27** schematic diagram of conversion from CF to CuO/CF


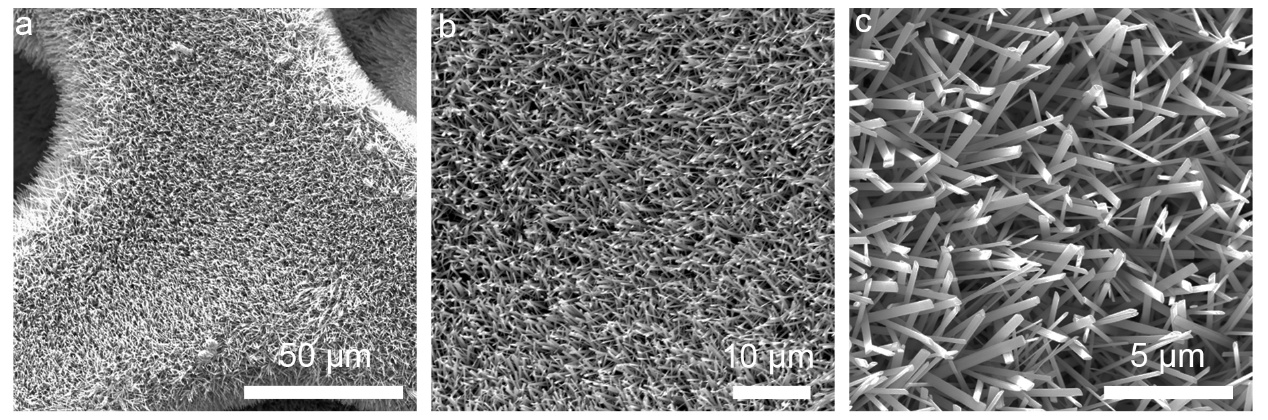


**Fig. S28** SEM images of Cu(OH)_2_


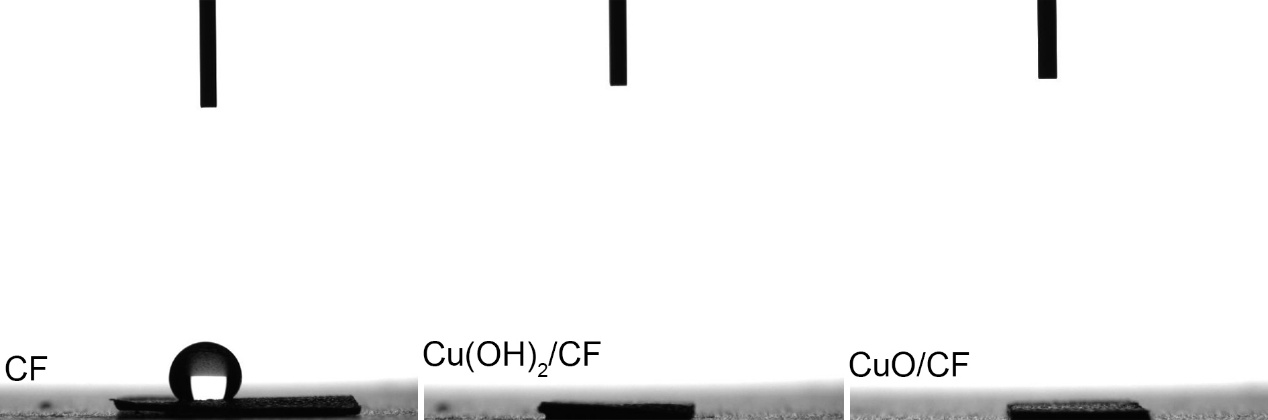


**Fig. S29** Contact angle of water droplets sitting on the surface of the **a**) CF, **b**) Cu(OH)_2_/CF, c) CuO/CF


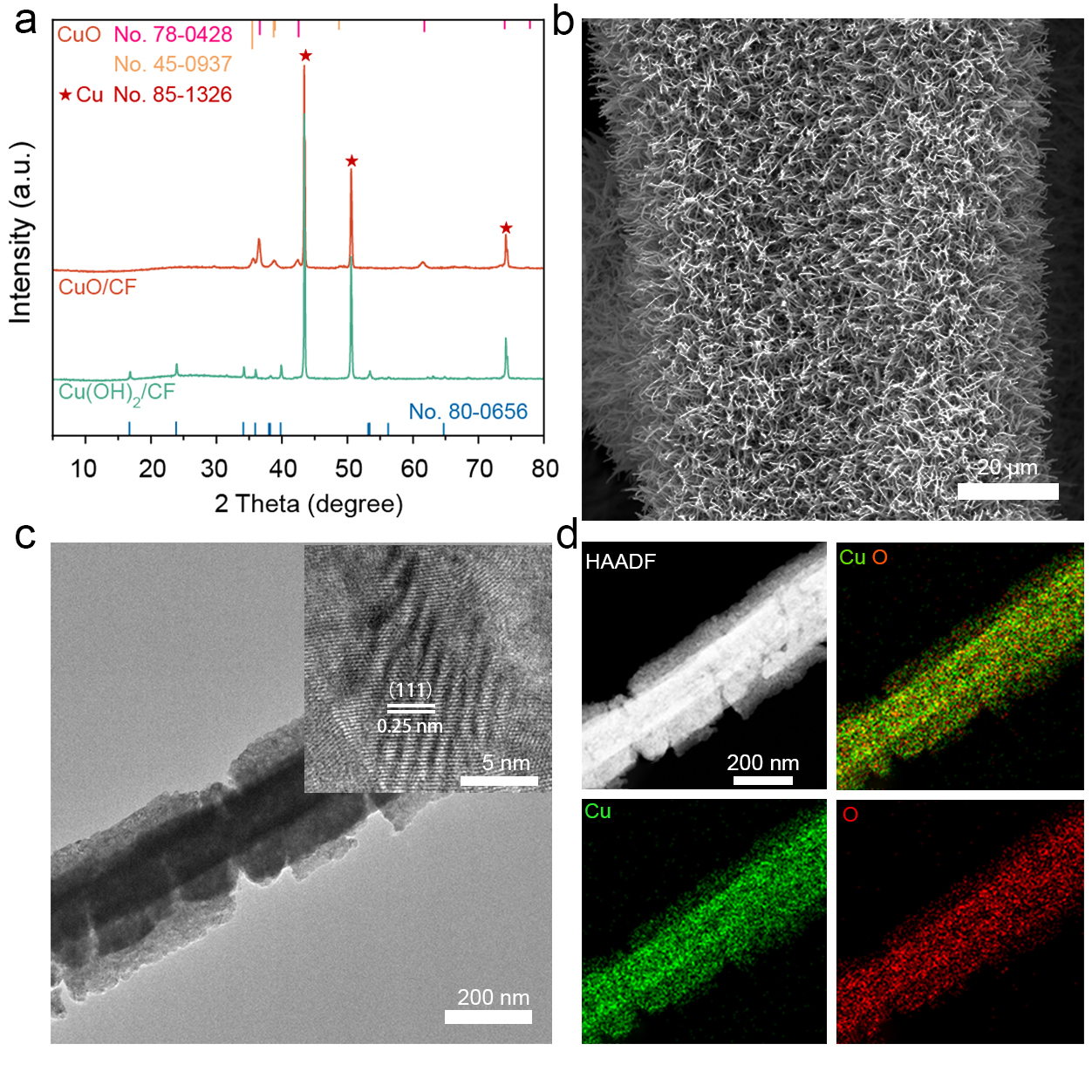


**Fig. S30** a) XRD patterns of CuO/CF and CuOH)_2_/CF. b) SEM and c) TEM images CuO of CuO/CF. c) Elemental mappings of Cu and O respectively, for CuO/CF


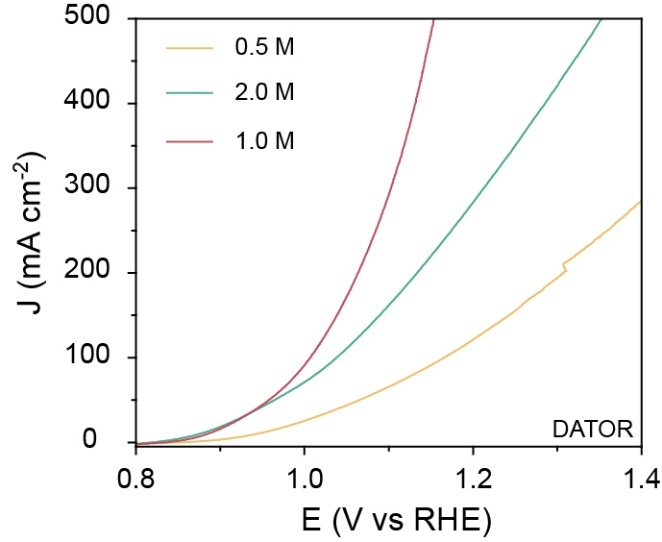


**Fig. S31** DATOR performance curves at different KOH concentrations


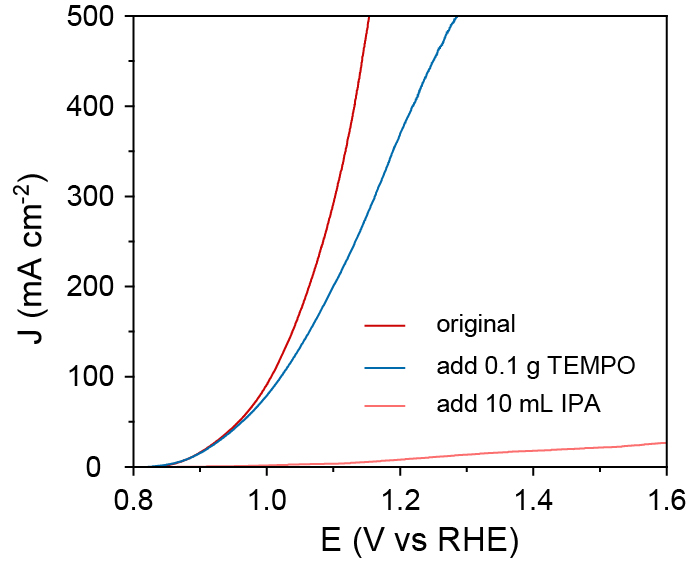


**Fig. S32** Performance curve of DATOR added with TEMPO and IPA


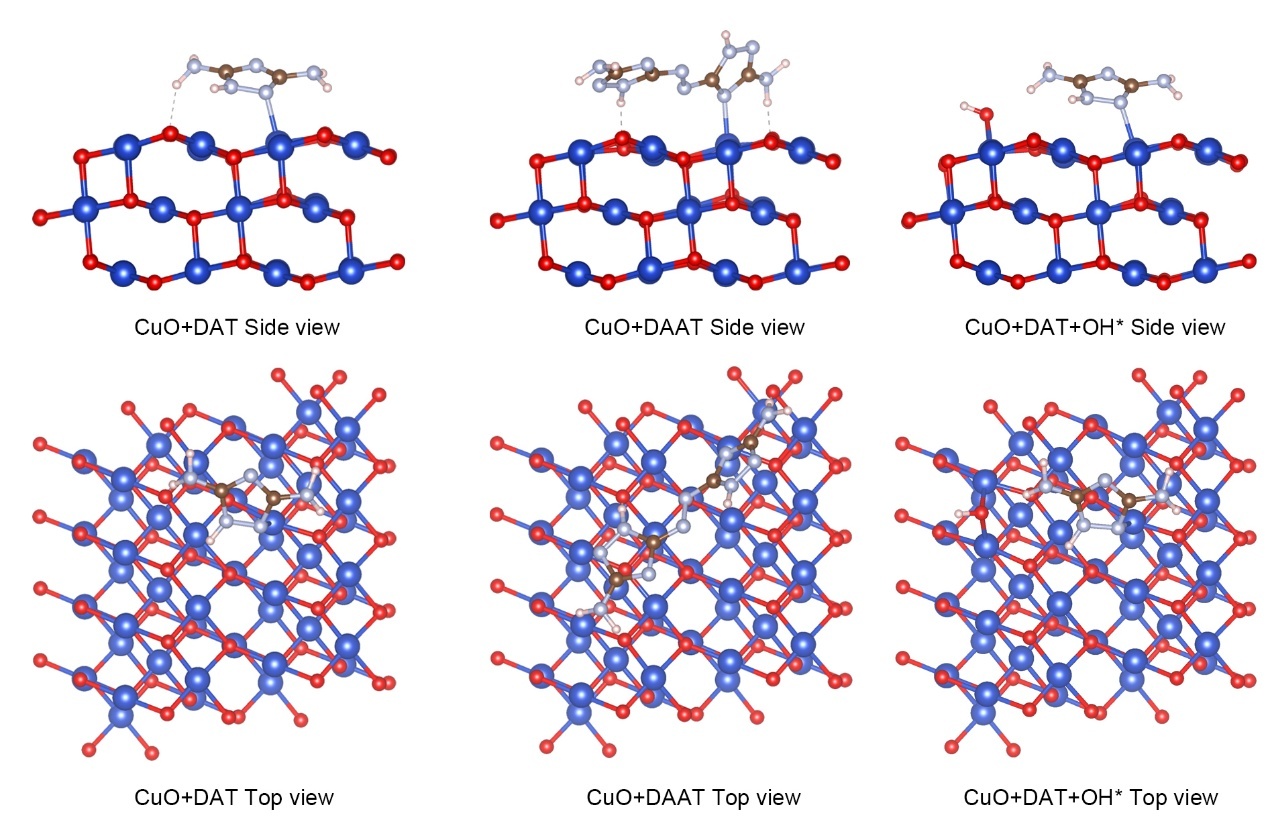


**Fig. S33** Theoretical model diagram for DAT, DAAT and DAAT+OH* load CuO


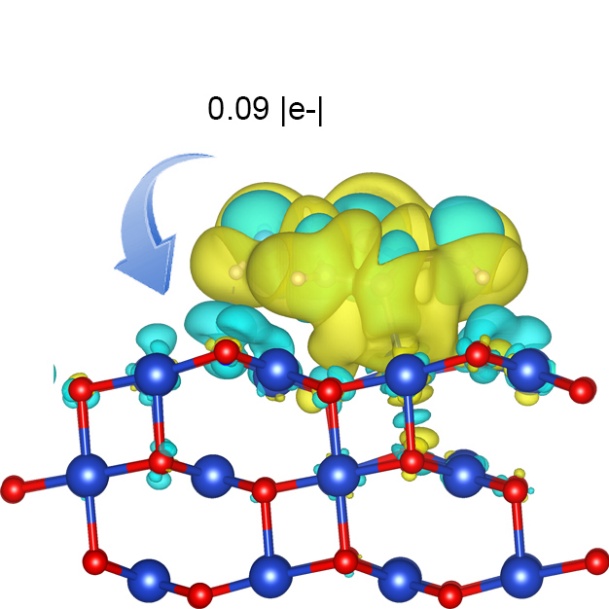


**Fig. S34** Differential charge density diagram of DAT on CuO surface


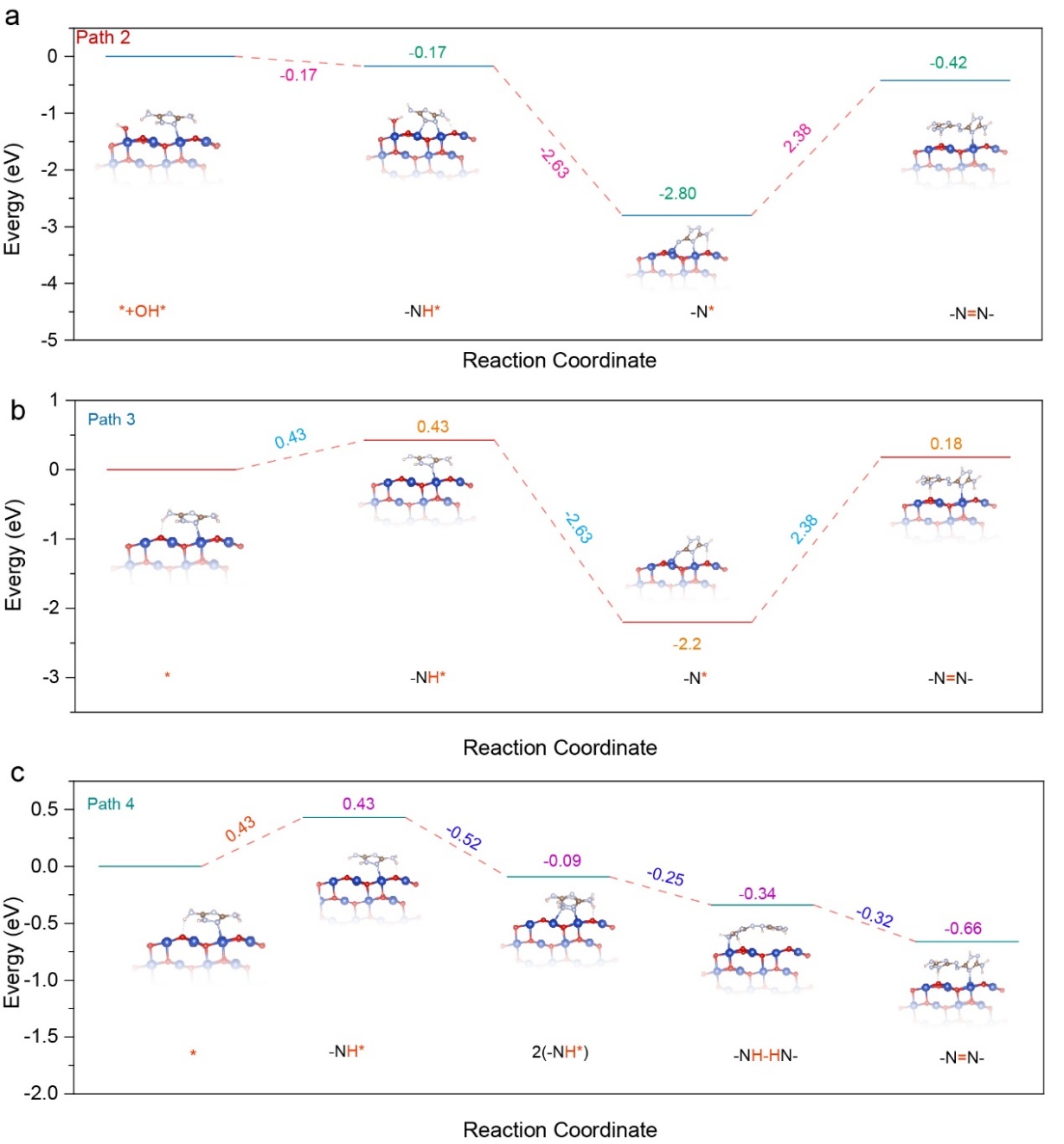


**Fig. S35** Mechanism diagram of With or without OH* induced DATOR (path 2-4)


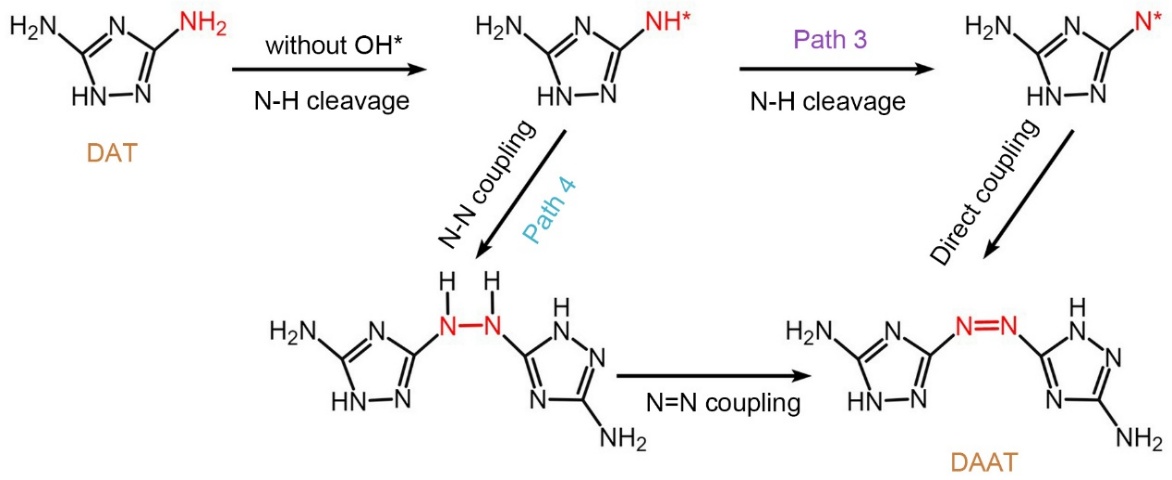


**Fig. S36** Mechanism diagram of without OH* induced DATOR (Path 3,4)


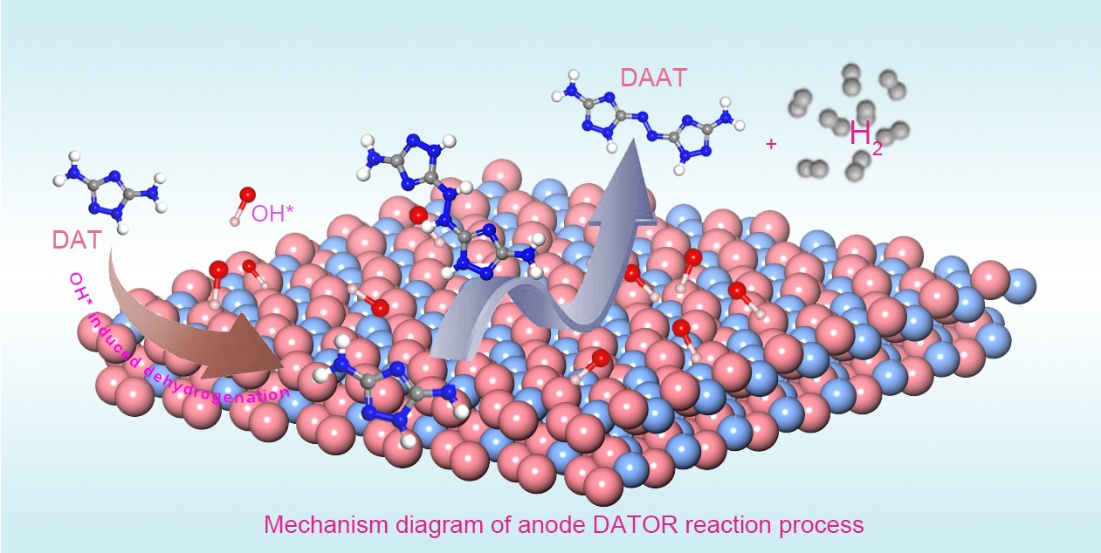


**Fig. S37** Illustration of possible synergistically catalytic mechanism of alkaline DATOR on CuO


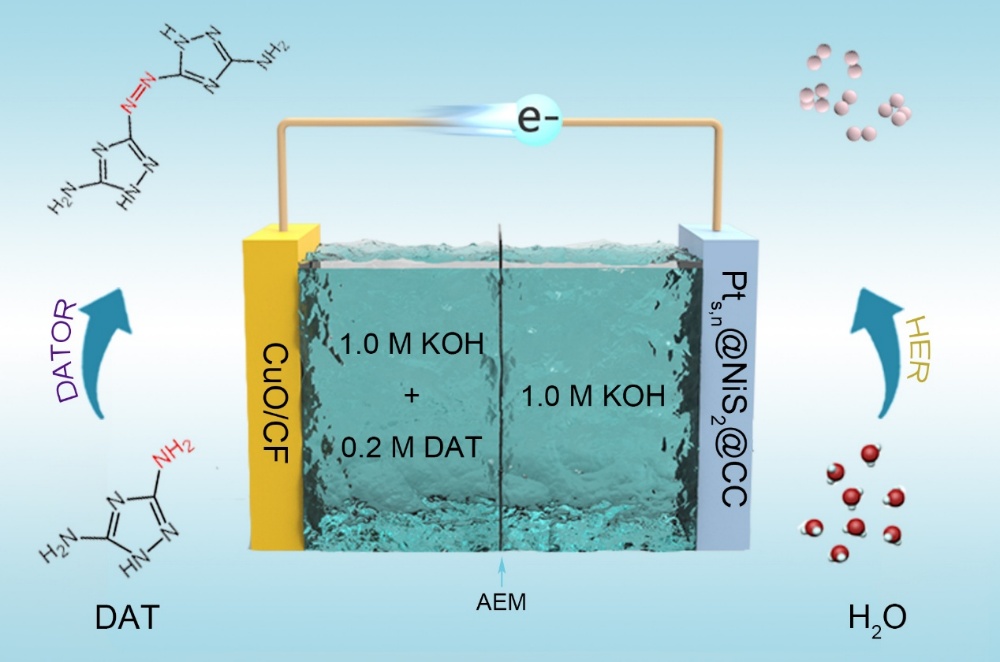


**Fig. S38** Schematic diagram of HER||DATOR coupling device


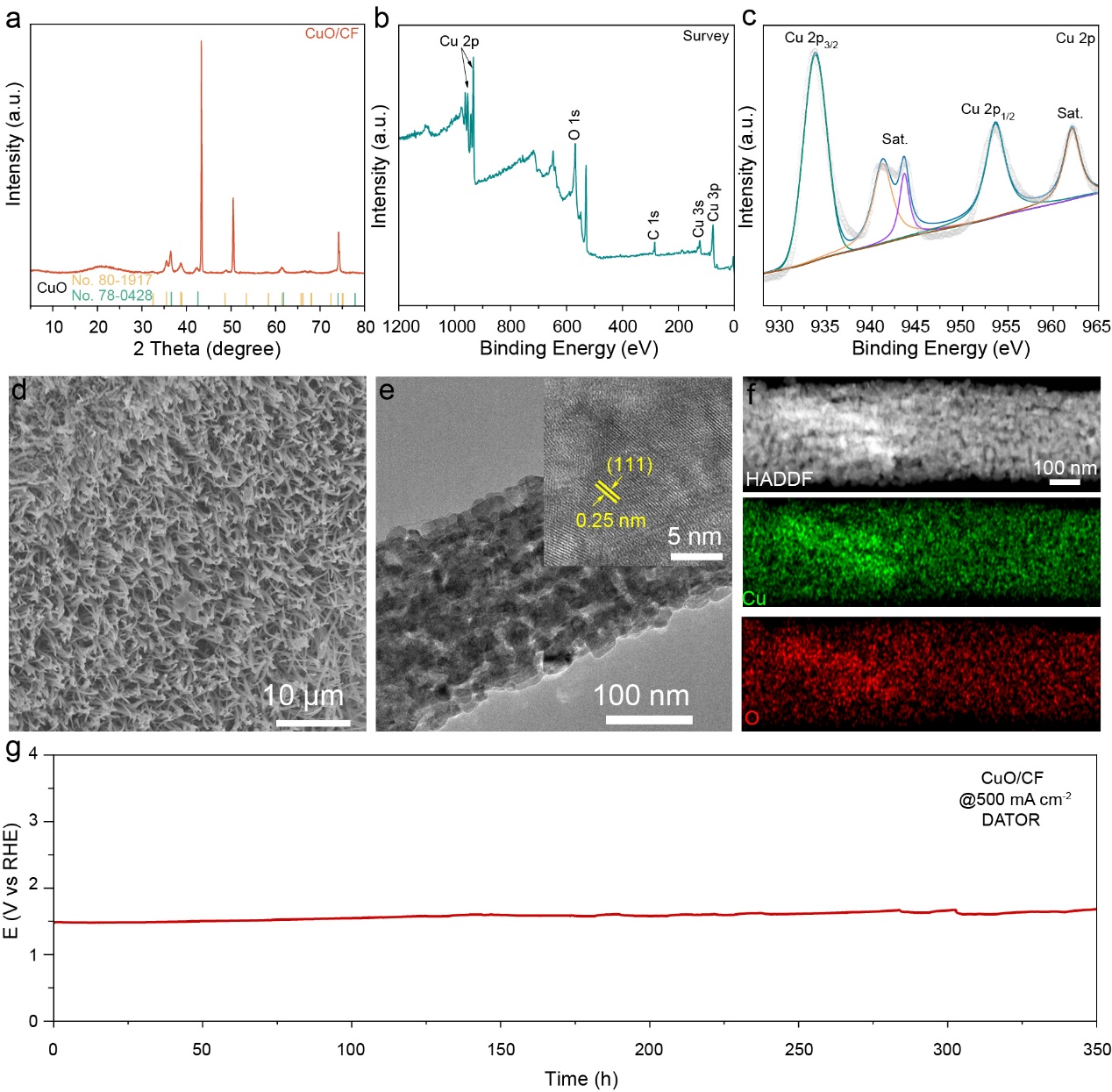


**Fig. S39** Structural and morphological characterizations of CuO/CF after the CP test. **a**) XRD patterns of CuO/CF. **b**) XPS survey spectrum and High-resolution spectra of **c**) Cu 2p signals for CuO/CF after CP test. **d**) SEM images of Pt_s,n_@NiS_2_@CC. **e**) Low and high-resolution TEM images of Pt_s,n_@NiS_2_@CC. **f**) Elemental mappings of Ni, Pt and S respectively, for Pt_s,n_@NiS_2_@CC. **g**) CP test of CuO/CF


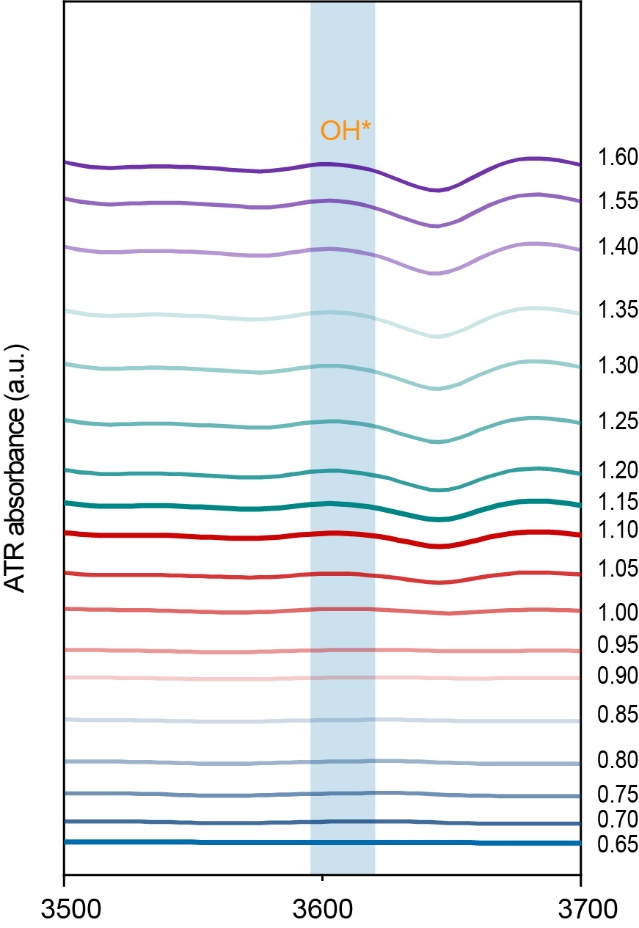


**Fig. S40** In-situ infrared spectrum of OH*


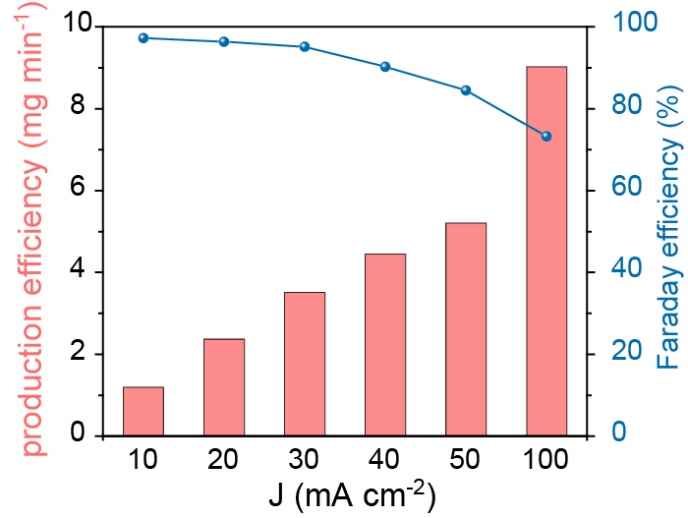


**Fig. S41** Faradaic efficiency of DATOR at various current densities

**Table S1** Comparison of OWS working potentials and HER performance for the Pt_s,n_@NiS_2_@CC at 10 mA cm^–2^ with those of recently reported state-of-the-art electrocatalysts

| Catalysts | η_10_ | Tafel | *Refs.* |
| --- | --- | --- | --- |
| Pt_s,n_@NiS_2_@CC | 36.7 | 33 | *This work* |
| Ru_3_Ni_3_NAS | 39 | 26.9 | *iscience,* ***2019****, 11, 492* |
| a-RuTe_2_PNRS | 36 | 36 | *Nat. Commun.,* ***2019****, 10, 5692* |
| Ni-BDT-A | 80 | 70 | *Nat. Commun.,* ***2017****, 8, 14580* |
| Er_2_Si_2_O_7_:IrO_2_ | 170 | 59 | *ACS Catal.,* ***2018****, 8, 8830* |
| Ir-Ni thin films | 60 | 40 | *Catal. Lett.,* ***2019****, 150, 1325* |
| IrCo@NC | 45 | 80 | *Adv. Mater.,* ***2018****, 30, 1705324* |
| Ni/Graphene | 29 | 87 | *Mater. Chem. And physics,* ***2021****, 272, 125049* |
| Ni-Mo-Cu | 49 | 62 | *Surf. Coat. Tech.,* ***2023****, 465, 129596* |
| Ni-Co alloys | 69.8 | 89.7 | *Int. J. Hydrog. Energy.,* ***2019****, 44, 29946* |
| Ni-Mo-S | 37 | 63.42 | *Int. J. Hydrog. Energy.,* ***2021****, 46, 3821* |
| Co-MoS_2_ | 58 | 100 | *Appl. surface. Sci.,* ***2020****, 516, 146094* |
| Co-Mn@Ni-Se | 22.1 | 45.05 | *Appl. Catal. B Environ.,* ***2023****, 325, 122355* |
| NiFe-LDH-Vo@NiRu | 24 | 92.4 | *Chem. Eng. J.,* ***2022****, 137226* |
| Ni_3_S_2_@NGCLS/NF | 134 | 99 | *Chem. Eng. J.,* ***2022****, 401, 126045* |
| NiS_2_/V-MXene | 179 | 85 | *J. Catal., 2019, 375, 8* |
| NiCoP/NF | 60 | 51.4 | *J. power sources.,* ***2021****, 484, 229269* |
| Ni-NiCoP_x_/NCF | 23 | 30 | *Chem. Eng. J.,* ***2020****, 402, 126257* |
| NiCo_2_O_4_@Ni_2_P | 141 | 86 | *Mater. Today. Energy.,* ***2020****, 17, 100490* |
| P Vo NiCo_2_O_4_ | 175 | 55 | *J. Power Sourece.,* ***2021****, 490, 229541* |
| CoSe_2_/N-CNT | 84 | 62 | *ACS. Appl. Mater. Interface.,* ***2019****, 11, 3372* |
| NiCoN | 48 | 78.7 | *J. Mater. Chem. A,* ***2019****, 7, 19728* |
| FeOOH@Co_4_N | 92 | 34 | *ACS. Appl. Mater. Interface.,* ***2019****, 11, 5152* |
| NiS_2_/CoS_2_/C | 165 | 72 | *Nano. Energy.,* ***2021****, 105940* |
| Ru/Co_3_O_4_ | 30.96 | 57.87 | *Chem. Commun.,* ***2019****, 55, 3781* |
| Cr_0.2_Ni_0.8_ | 106 | 71 | *Int. J. Hydrog. Energy,* ***2020****, 45, 17533* |
| Cr-doped cop nanorods | 36 | 54 | *Cell Rep. Phys. Sci.,* ***2020****, 100136* |
| PtSA-NiSe-V | 45 | 52 | *Angew. Chem. Int. Ed,* ***2023.*** |
| Ni_2_P/CoP-Pt | 44.5 | 58.4 | *Adv. Funct. Mater.,* ***2023.*** |
| Pt-Ni(OH)_X_ | 58 | 84 | *Adv. Energy. Mater.,* ***2023.*** |
| Ni_2_P-Ni_12_P_5_@Ni_3_S_2_/NF | 34 | 146 | *Adv. Mater.,* ***2022.*** |
| PtSA-Ni_3_Fe LDH | 45 | 37.8 | *Energ. Environ. Sci.,* ***2021.*** |
| PtNi_10_/C | 66 | 16.73 | *J. Colloid Interface Sci. ,* ***2023****, 634, 897-905* |
| Mixture phases PtFe | 28 | 42 | *Small.,* ***2022****, 18, 2106947* |
| PtFeNiCuCo | 27 | 34.7 | *J. phys.Conf. Ser.,* ***2022****, 2254, 012006* |
| Pt/NiCo@C | 48 | 130 | *Electrochimica Acta.,* ***2023****, 460, 142634* |
| Ni-MOF@Pt | 102 | 88 | *Nano Lett.,* ***2019****, 19, 8447* |
| Ni_3_S_2_@20 Co-MOF/CP | 140 | 90.3 | *Fuel Process. Technol.,* ***2022****, 229, 107174* |
| CoO/N-S-UPCNPs-600 | 120 | 94 | *Chem. Electro. Chem.,* ***2019****, 6, 3940* |

**Table S2** Comparison of potential at 10 mA cm^–2^ for anodic oxidation reaction to upgrade chemicals with recently reported hybrid water electrolysis. (HMF: 5-Hydroxymethylfurfural)

| Catalysts | Substrate | Potential(E_10_) | *Ref.* |
| --- | --- | --- | --- |
| CuO/CF | DAT | 0.96 | *This work* |
| Mo-Co_4_N | Methanol | 1.427 | *J. Mater. Chem. A,* ***2021****, 9, 21094* |
| Co(OH)_2_@HOS/CP | Methanol | 1.497 | *Adv. Funct. Mater.,* ***2020****, 30, 1909610* |
| CoCu-bi-MOF | Methanol | 1.365 | *Appl. mater. lnterf.,* ***2018****, 10, 25422* |
| FeRu-MOF | Methanol | 1.32 | *esci.,* ***2023****, 100118* |
| NiIr-MOF/NF | Methanol | 1.33 | *Appl. Catal. B-environ.,* ***2022****, 121667* |
| 4MOFNs | Methanol | 1.365 | *J. Colloid. lnterf. Sci,* ***2022****, 616, 279* |
| Ni_2_P/NiMoP | urea | 1.35 | *Esci.,* ***2021****, 1, 69* |
| Ni_3_N/N_0.2_Mo_0.8_N | urea | 1.348 | *Chem. Eng. J.,* ***2021****, 409, 128240* |
| Ni-Mo-O | urea | 1.38 | *Energ. Environ. Sci.,* ***2018****, 8, 1-7* |
| Ni_2_Fe(CN)_6_ | urea | 1.38 | *Nat. Energ.,* ***2021****, 6, 904* |
| CoS_2_/MoS_2_ | urea | 1.29 | *Adv. Funct. Mater.,* ***2018****, 8, 1801775* |
| NiMoO_4_@NiFeP | urea | 1.30 | *Adv. Energ.Mater.,* ***2019****, 9, 1900390* |
| Ni-Mo-N | Glycerol | 1.36 | *Nat. Commun,* ***2019****, 10, 5335* |
| HEA-CoNiCuMnMo | Glycerol | 1.34 | *J. Am.Chem. Soc.,* ***2022****, 144, 7224* |
| MnO_2_ | Glycerol | 1.38 | *Angew. Chem. Int. Ed.,* ***2021****, 133, 21634* |
| Rh_x_[Ni(OH)_2_]_y_/C | Glycerol | 1.29 | *Int. J. Hydrogen. Energ.,* ***2023****, 48, 31091* |
| Ru-Ni_x_P_y_/N-C | Glycerol | 1.36 | *Ind. Eng. Chem. Res.,* ***2019****, 58, 16077* |
| Ru@Ni-B/NF | Glycerol | 1.24 | *Chem. Rev.,* ***2019****,120, 526* |
| MoO_2_-FeP | HMF | 1.486 | *Adv. Mater.,* ***2020****, 32, 2000455* |
| Co-CoS_x_@CN | HMF | 1.29 | *Chem. sci.,* ***2022****, 13, 4647* |
| Ni(OH)_2_/NF | HMF | 1.39 | *Chem. Suschem.,* ***2021****, 14, 2935* |
| δ-MnO_2_/NF | HMF | 1.35 | *Chem. Mater.,* ***2022****, 34, 3123* |
| CuCoO_4_/NF | HMF | 1.23 | *Angew. Chem. Int. Ed.,* ***2020****, 33, 385* |
| W-doped Ni_3_S_2_@NF | HMF | 1.34 | *Sci. China. Chem.,* ***2023****, 66, 3636* |
